# New Insights into the Key Role of Thermal Treatment in V/P/O Catalysts for the Selective Oxidation of *n*-Butane to Maleic Anhydride

**DOI:** 10.1021/acsomega.5c00501

**Published:** 2025-02-11

**Authors:** Ludovica Conte, Laura Setti, Giacomo Luzzati, Tommaso Tabanelli, Laura Fratalocchi, Lorenzo Grazia, Silvia Luciani, Silvia Bordoni, Carlotta Cortelli, Fabrizio Cavani

**Affiliations:** † Dipartimento di Chimica Industriale “Toso Montanari”, Università di Bologna, Viale Risorgimento 4, Bologna 40136, Italy; ‡ Center for Chemical Catalysis—C^3^, Alma Mater Studiorum Università di Bologna, Viale Risorgimento 4, Bologna 40136, Italy; § Polynt SpA, Via E. Fermi 51, 24020 Scanzorosciate, BG, Italy

## Abstract

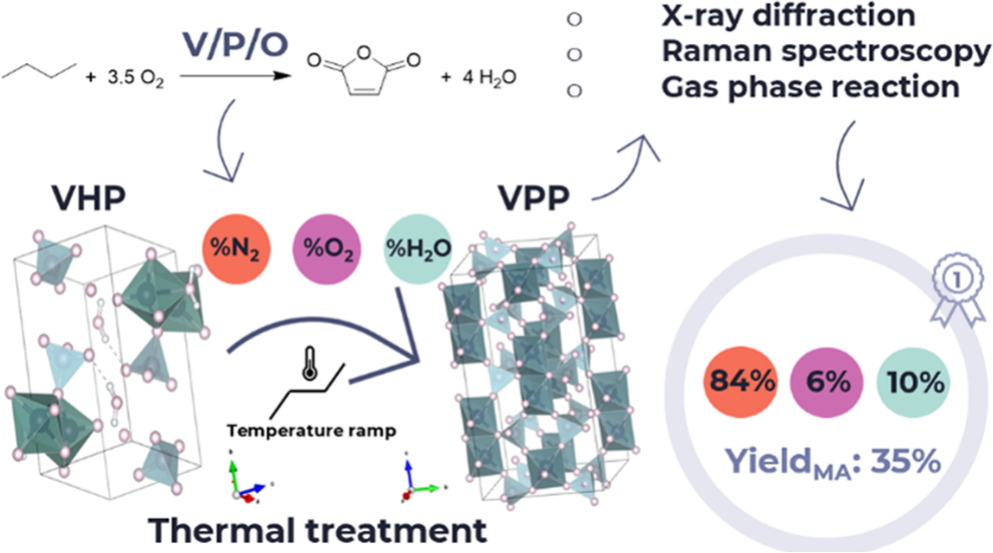

This work explores the thermal treatment of V/P/O catalyst precursors to achieve active and selective catalysts for the oxidation of *n*-butane to maleic anhydride (MA) in a continuous-flow fixed-bed reactor. Vanadyl pyrophosphate (V^4+^, VPP), the key catalyst component, is produced together with suitable V^5+^ vanadium orthophosphate (VOPO_4_) allotropic forms by thermally treating vanadyl hydrogen phosphate hemihydrate (VHP) under various atmospheres and temperature ramps. The characterization conducted by using X-ray diffraction, Raman spectroscopy, and reaction testing allowed the identification of optimal conditions for active and selective catalysts. Oxygen is necessary for obtaining VPP and affects the vanadium oxidation state, which is a crucial parameter for selectivity. Water enhances the crystallinity and conversion of VHP to VPP. An optimized calcination atmosphere (6:10:84 mol % O_2_/H_2_O/N_2_) ensures 70% MA selectivity at 50% butane conversion at 400 °C. VHP precursors characterized by higher P/V ratios allow us to obtain higher MA selectivity when treated under the same calcination conditions. This study provides valuable insights into the VPP production steps, representing the starting point for fine-tuning the calcination conditions based on the VHP properties (i.e., P/V ratio and carbon content).

## Introduction

Maleic anhydride (MA) is a versatile molecule due to its three active sites, i.e., two carboxylic groups and a double carbon bond. It is considered an important building block in industrial chemistry, with several applications such as monomers for polyesters and alkyd resins, as well as an intermediate to produce fine chemicals (succinic acid, malic acid, and fumaric acid).^1^ MA production volume worldwide is around 3 Mtons/year, with China as a major global producer.^1,2^

The major process for MA production is the selective oxidation of *n*-butane, with vanadyl pyrophosphate (VO)_2_P_2_O_7_ (VPP) as the main catalyst component.^3,4^ Its synthesis is widely described in the literature.^5−8^ Briefly, vanadyl hydrogen phosphate hemihydrate (VHP, the precursor of the final material) is usually prepared using an organic medium, i.e., alcohol (commonly isobutanol) or a mixture of alcohols, acting both as a reducing agent and as a solvent. V_2_O_5_ is dissolved by the formation of a vanadyl alcoholate and reduced to V^4+^, and then H_3_PO_4_ is added to the mixture, leading to the formation of crystalline VHP. The solid is recovered by filtration, centrifugation, and dehydration at 300 °C to remove organic compounds from the synthesis. The Polynt Group S.p.A. also used this synthetic path, which supplied the precursor for the present study.

**Figure 1:**
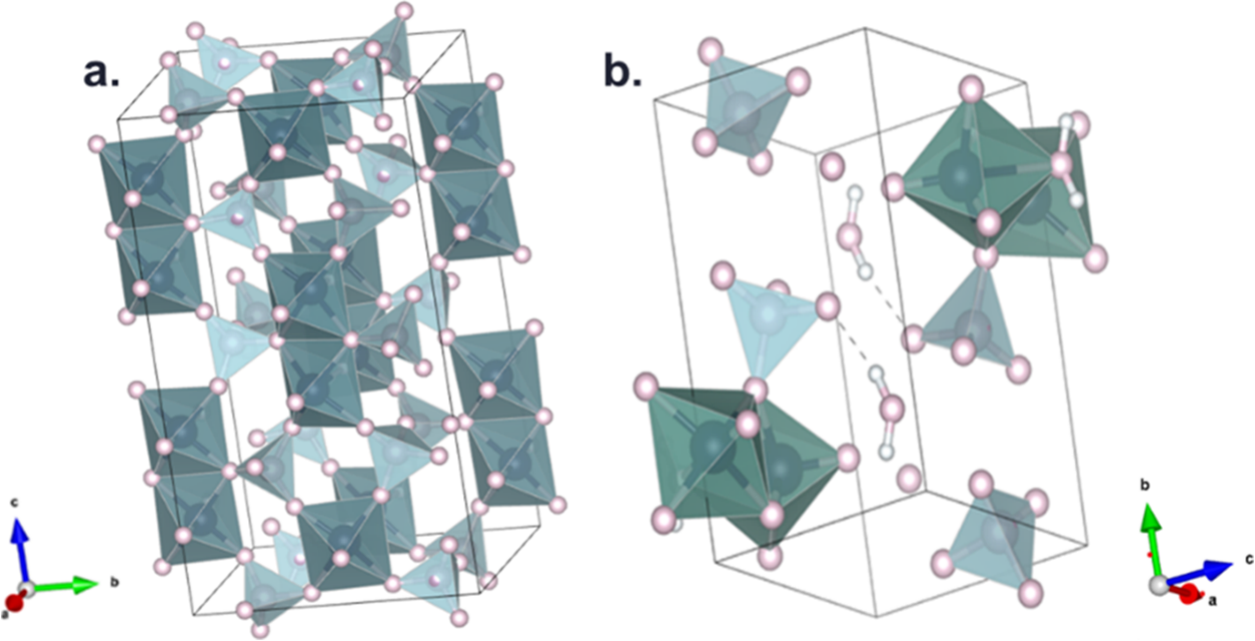
Crystalline structure of VPP (a) and VHP (b).^12^

After dehydration, the VHP precursor undergoes heat treatment under a controlled atmosphere to yield the active phase. Indeed, VHP presents a structure composed of face-shared octahedra [VO_6_] linked by hydrogen phosphate tetrahedral groups, with a water molecule bridge connected between two adjacent vanadium atoms and O–H groups in the trans position, concerning the V=O bond.^9^ VPP is formed during the treatment via a topotactic transformation^10,11^ in which V–O and V–P bonds remain unaltered. At the same time, the weak V–H_2_O and P–H_2_O decompose, leading to a new type of edge-shared octahedra, together with a proton transfer from two vicinal P–OH groups, decreasing the distance between layers and forming pyrophosphate groups (Figure 1).

After heat treatment, the obtained material contains vanadyl pyrophosphate (VPP, (VO)_2_P_2_O_7_) as the main phase and V^5+^ vanadium orthophosphate (VOPO_4_) compounds. VPP is a V^4+^ phosphate in three crystalline forms: α, β, and γ.^13,14^ These polymorphs can be easily identified through X-ray diffraction (XRD) analysis based on the varying intensities of the characteristic diffraction line centered at 2θ = 23°, corresponding to the 200 crystallographic planes. β-VPP is reported to be highly active but with limited selectivity to MA, while α and γ-VPP usually present a sharp 200 peak and possess moderate activity but higher selectivity for MA.^15,16^

VOPO_4_ can be present in different forms, namely, α_I_, α_II_, β, δ, γ, ω and the hydrated phases VOPO_4_·*n*H_2_O.^17,18^ Notably, these oxidized VOPO_4_ compounds play a major role in fine-tuning the catalytic activity of VPP, thus driving the selectivity for the selective oxidation to MA. In the literature, it has been reported that δ-VOPO_4_ and γ-VOPO_4_ present lower activities but higher selectivity to MA; α phases, on the other hand, exhibit higher activities but are unselective for the target reaction.^17^ ω-VOPO_4_ has a structure similar to that of δ and can be converted under reaction conditions.^19^ β-VOPO_4_ is the most stable phase and does not present any selectivity, resulting in being highly undesired.^15^

In ongoing research aimed at fine-tuning the catalytic properties of V/P/O catalysts and optimizing their activity for the target reaction, some crucial parameters must be considered, such as the P/V atomic ratio, average V oxidation state, and catalyst acidity. The former influences the selectivity of the target reaction: the reason for the different catalytic behavior lies in the preferential formation of VOPO_4_ phases depending on the P/V ratio; if it is greater than one, the formation of the highly selective δ-VOPO_4_ is favored; if the ratio is lower than one, undesired α_I_-VOPO_4_ will form during the reaction. Regarding the overall V oxidation state, it was demonstrated that the optimal value for V^5+^/V^4+^ is close to 0.25, a condition in which the catalyst presents a limited amount of VOPO_4_ over a VPP core.^20^ On the other hand, an excess of V^5+^ is detrimental to the catalytic performances. Concerning acidity, VPP contains both Lewis and Brønsted acid sites, which play different roles in the reaction mechanism.

The cited key properties can be easily tuned with a controlled calcination process. Until now, few studies in the literature have covered the important matter of the structure–activity correlation derived from the calcination atmosphere. A pivotal work in this area was reported by Zhang et al.^21^ The authors found that by controlling the steam content during the activation process, it is possible to modulate the catalytic behavior of the final catalyst. Specifically, a moderate amount of steam played a role in stabilizing the V^5+^–O–P=O sites. The authors also provided an interpretation of the role of steam in affecting the nature of the active sites involved in the mechanism of *n*-butane oxidation. It was reported that calcination at 440 °C for 10 h with optimized water content (27% in the calcination mixture with air) produces high-performing catalysts with an ideal V^4+^/V^5+^ ratio, crucial for ensuring stable performance.^21^ Additionally, a study by Zhang et al. demonstrates that adjusting the air/nitrogen ratio during thermal treatment at 220 °C for 8h, followed by activation with 50% water at 395 °C for 8h, influences the catalyst’s oxidation state and the formation of amorphous phases and consequently the catalytic activity.^22^

Starting from literature findings on the role of steam during the precursor thermal treatment and on preliminary results that we recently patented,^23^ the scope of the present study is to further investigate the role of the conditions of the thermal treatment (activation) procedure on the VPP-based catalyst. Distinguished features of the present study, with respect to the literature already available on this topic, are (a) an investigation of how the properties of the catalyst precursor, the latter being affected by the procedure of preparation used, are affected by the composition of the gas phase used for the thermal treatment. Two industrial precursors for the fluid-bed reactor delivered by Polynt S.p.A were used for this study, differing in the P/V ratio, residual C content, and vanadium oxidation state. (b) An in-depth investigation of phenomena occurring during the thermal treatment of the precursor by an analysis of gaseous molecules released during the crystalline transformation of the precursor.

The in situ monitoring of the compounds released during the thermal treatment allowed us to understand that steam plays an important role in the removal of the organic residues still present in the precursor, thus facilitating the crystallization of VPP. Therefore, the presence of steam during activation is fundamental, with catalyst precursors showing a greater amount of residual organic compounds retained in the crystalline structure. Moreover, experimental evidence was obtained highlighting the role of steam in affecting the nature of the VPP polytype formed. The present study allowed us to understand that thermal treatment must be designed as a function of the characteristics of the catalyst precursor.

From a broader perspective, this work aims to contribute to the calcination process optimization for industrial precursors by customizing the procedure based on the specific characteristics derived from the synthetic process.

## Experimental Section

VPP catalysts were prepared by varying the heat treatment conditions starting from two VHP (vanadyl hydrogen phosphate hemihydrate, VOHPO_4_·0.5H_2_O) precursors provided by the Polynt Group S.p.A. The two precursors have diverse properties of residual carbon content, surface area, overall vanadium oxidation state, and P/V ratio, as described in the Results and Discussion section. Thermal treatments were conducted in a lab-scale, continuous-flow, fixed-bed glass reactor. Various compounds (air, O_2_, and N_2_) can be fed with steam to control the atmosphere during the temperature ramp or the isothermal steps, keeping the system at atmospheric pressure. The outgoing flows were analyzed with an online microgas chromatograph Agilent 3000 equipped with an HP Plot-Q column, OV1, and a Molecular sieve 5 Å column, used to separate and quantify water, carbon dioxide, and oxygen and detect their concentrations using a thermal conductivity detector (TCD). A schematic of the plant used for the heat treatments is reported in the SI in Figure S1. In a typical heat treatment, 2 g of the precursor powder was heated from RT to 180 °C at 2 °C/min in air (STEP 1). After that, it was heated to 425 °C at the same rate, with a following 2 h isothermal step, in a mixture having a different composition depending on the calcination conditions (STEP 2); finally, another temperature ramp (2 °C/min) was used up to 500 °C and finally kept for 2 h in N_2_ (STEP 3). The contact time was fixed at 0.065 g·s/mL, regardless of the calcination mixtures used.

All of the *n*-butane selective oxidation tests were carried out in another dedicated lab-scale, continuous-flow, fixed-bed glass reactor controlling different parameters such as feed composition, contact time, and temperature (Figure S2). The outlet gaseous stream was split and analyzed using an online GC equipped with both a flame ionization detector (FID), with a CpSil-5CB column used for the quantification of hydrocarbons, acids, and MA; and a TCD with a Packed Carbosieve SII column (80–100 mesh) for the quantification of the incondensable products. A dedicated trap was inserted before the line dedicated to TCD-GC analysis, allowing the condensation of MA and heavy compounds.

In a typical catalytic test, a mixture with %mol composition equal to 1.7:17:81.3 *n*-butane:oxygen:inert (mixture of N_2_ and He) is fed to the reactor with a W/F of 1.33 g·s·mL^–1^, with the catalysts shaped in the form of 30–40 mesh pellets. This mixture is necessary to avoid explosion hazards (the lower and upper flammability limits of *n*-butane are equal to 1.8 and 8.4 mol %, respectively). A 50 h equilibration step is conducted by maintaining the catalyst under the reaction mixture flow at 400 °C to achieve steady catalytic performance. Finally, the catalytic experiments are carried out at three different temperatures of 400, 420, and 440 °C. The carbon balances of all the catalytic tests were between 96 and 100%.

To characterize the structural properties of the materials, X-ray diffraction was used to determine the crystalline structures of the VPP catalysts using Cu Kα radiation (λ = 1.54178 Å, Ni-filtered) on a Philips X’Pert vertical diffractometer in Bragg–Brentano geometry, featuring a pulse height analyzer and a secondary curved graphite-crystal monochromator, in the range of 5 < 2θ < 90° with a scanning rate of 0.05°/s and a time per step of 1 s. The specific surface area was determined by the physisorption of liquid nitrogen at 77 K using a Micromeritics ASAP 2420 instrument using the Brunauer–Emmett–Teller (BET) equation. Thermogravimetric analysis (TGA) was conducted to quantify the weight loss as a function of time and temperature caused by adsorbed molecules on the samples with a DST Q600 TA Instrument (either in air or N_2_ flow). The surficial properties were evaluated using Raman spectroscopy and energy-dispersive X-ray spectroscopy. The first, with the aim of screening the phase on the surface of the catalyst, was performed with a Renishaw Raman System, RM1000 with Leica DLML confocal microscope with 5×, 20×, and 50× objectives, and a laser source argon ion (514 nm) with a power of 25 mW. The analysis was conducted by pointing to several regions of possible interest (different colors, shapes, etc.) on the catalyst surface, with the following parameters: 5 accumulation, 10 s, 10% laser power to prevent sample damage, and 50× objective. The Raman peaks used for compound identification are listed in Table S1.

The composition in terms of the P/V ratio of the samples was investigated through X-ray fluorescence (XRF) analysis, performed using a Bruker S6 Jaguar by grinding and mixing the sample with a ligand at a pressure of 10 tons. The carbon content in the precursors was determined by combustion and nondispersive infrared detection by placing a weighted amount of the sample under oxygen flow at 1350 °C and measuring the carbon dioxide concentration with a LECO SC-144 DR instrument. Using a software equation that considers sample weight and calibration, the instrument converts these values to a percentage/ppm value. Finally, redox titration was performed, as reported in the ESI (P1), to determine the overall oxidation state of vanadium. Scanning electron microscopy-energy-dispersive X-ray spectroscopy (SEM-EDX) analyses were carried out with an environmental scanning electron microscope (E-SEM, Zeiss EP EVO 50) equipped with an energy-dispersive spectroscopy (EDS) microprobe (Oxford Instruments INCA X-act Penta FET Precision[*z* > 4 (Be), resolution 129 eV (MnKa @ 2500cps)]) with INCA Microanalysis Suite Software (version 4.15- issue.18 service pack 5), at EHT 20 keV unde high vacuum (∼10^–4^Pa) conditions. Punctual analyses and elemental distribution maps were obtained at different magnifications.

Table 1 summarizes all samples considered in this work. The samples are reported with their code “OX-LY”, in which *X* represents the % mol of oxygen (“O”) and *Y* the % mol of water (“L”, if used), used during STEP 2, from 180 to 425 °C + 2 h isothermal step.

**Table 1:**
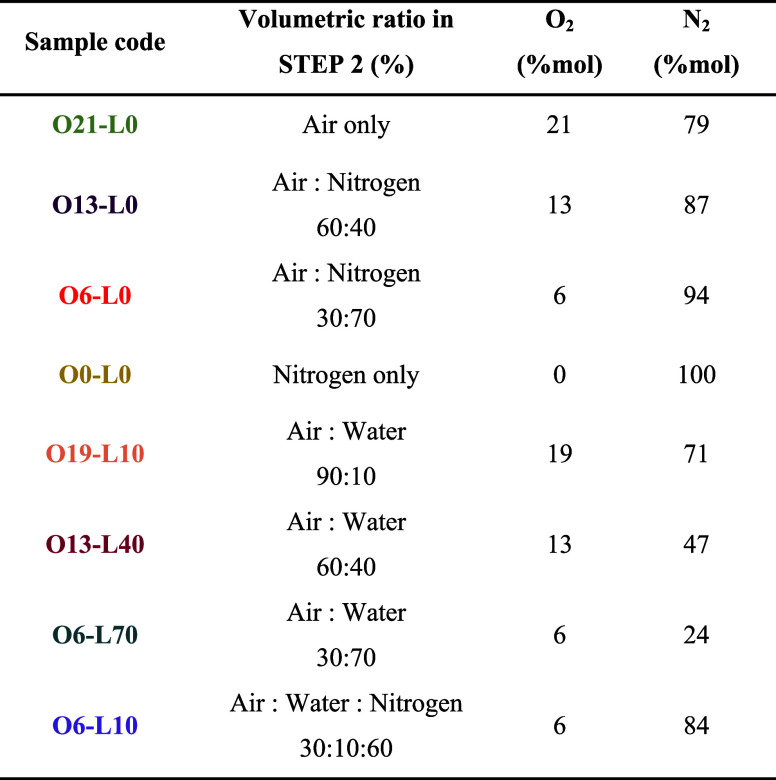
Resume of All the Samples Calcined for this Work, with STEP 2 Calcination Mixture Information in Detail (Water in the Vapor Form)^a^

a Precursor M17.

Diffuse-reflectance-infrared-Fourier-transform spectroscopy (DRIFT) was used to verify the surface acid properties of the thermally treated catalysts. Typically, the sample was diluted in KBr (50 wt %), loaded into the cell, and pretreated at 400 °C under a flow of He (10 mL/min) and air (10 mL/min) for 60 min to clean the surface and remove any adsorbed molecules. Then, using KBr as the background, the spectra were acquired at 50 °C. Next, a pulse of pyridine (1 μL) was introduced into the sample at 50 °C. The sample was flushed for 1 h with He before collecting the IR spectra in situ with a Bruker Vertex 70 instrument equipped with a Pike DiffusIR cell attachment. Spectra were recorded using an MCT detector after 128 scans with a 4 cm^–1^ resolution in the 4000–450 cm^–1^ region. The Brønsted to Lewis ratio (B/L) was estimated, as previously reported by Cornaglia et al.^33^

## Results and Discussion

### Characterization of Precursors and Preliminary Investigation of the Role of Calcination Atmosphere

Two industrial VHP precursors (coded as M16 and M17) were characterized by X-ray fluorescence analysis to determine the P/V atomic ratio, as reported in Table 2, highlighting that M17 has a lower P/V than M16. This could lead to variations in their catalytic performance since an excess of P is required to improve the MA selectivity. Moreover, the value of the residual carbonaceous compound derived from the synthesis is lower for precursor M16. The V^5+^ content, as determined by titration, is lower in M17 than in M16. Their XRD patterns (Figure S3) show the characteristic diffraction lines of crystalline VOHPO_4_·0.5H_2_O. Notably, the intensity ratio of the two main peaks at 15.5 and 30.4° 2θ, related, respectively, to the (010) and (202) crystallographic planes, are different: M17 presents a lower value for the *I*_202_/*I*_010_ ratio and is expected to show a lower selectivity to MA, as reported in the literature.^24^ The surface area is higher for M16, probably because it presents a lower crystallinity than M17.

**Table 2:** Characterization of VHP Precursors

precursor	P/V^a^	*C*_in_^b^ (ppm)	%V^5+^^c^	SSA^d^ (m^2^/g)
M17	1.15	9490	0.006	12
M16	1.20	6344	0.040	17

a P/V atomic ratio obtained by XRF with an associated error of 0.3%.

b C content obtained from combustion analysis.

c Percentage of V^5+^ obtained from titration.

d Surface area obtained from the nitrogen porosimeter with an associated error of 3%.

Some preliminary calcination tests were conducted with M17, both in the lab-scale reactor and in the TGA instrument, to rapidly assess the effect of different atmospheres on compound evolution under anhydrous conditions. In particular, three different calcination atmospheres were tested: (a) air flow with a switch to nitrogen at 425 °C, (b) only nitrogen, and (c) only air. In all cases, calcination was conducted with a ramp rate of 2 °C/min from room temperature to 500 °C, lasting 8 h. Several important considerations can be highlighted through weight loss analysis and the evolution of water and carbon dioxide (followed by an online micro-GC). When only N_2_ was used, the sample showed the highest weight loss and lowest overall V oxidation number (Table 3). This is due to the combustion of the organic residues from the synthesis promoted by the V sites at the expense of lattice oxygen. In contrast, the sample calcined by feeding only air can readily restore the V oxidation state, leading to the highest overall value of 4.66. Therefore the sample calcined with a mixed atmosphere (switch to N_2_ at 425 °C) presents intermediate weight loss and V oxidation state, with potential benefit in both activity and selectivity, as shown later.

**Table 3:** Average V Oxidation States^a^ Obtained from Titration and Weight Loss ^b^ after the Thermal Treatment (Obtained from TGA Analysis) of the M17 Precursor

case	atmosphere	Vox^a^	weight loss (%)^b^
a	RT → 425 °C air	4.52	12.3
	425 → 500 °C nitrogen		
b	nitrogen only	3.85	14.5
c	air only	4.66	11.2

All of the materials obtained were characterized using both XRD and Raman spectroscopy (Figure S4). In the case of treatment “a” (Table 3), the main compound observed was ω-VOPO_4_ (Raman bands at 1188, 1084, 1016, 932, 650, and 589 cm^–1^), with traces of VPP (1185 and 920 cm^–1^), and orthophosphate dihydrate (VOPO_4_·2H_2_O: 1039, 988, 952, and542 cm^–1^), probably present due to the coproduced water remaining trapped in the crystalline structure. On the other hand, the heat treatment performed completely in air (case c) clearly led to an overoxidized material in which VPP was the minor compound (see the XRD pattern, Figure S4). In the Raman spectrum, ω-VOPO_4_ was the main component with a total absence of VPP. On the contrary, when only nitrogen was fed during the treatment (case b), the XRD pattern showed reflections typical of the VPP phase. Nevertheless, the Raman spectrum revealed the presence of residual carbonaceous species because, as expected, the vanadium ion oxidizing action was insufficient to promote complete combustion of the organic species derived from the synthesis. For all the cited reasons, we continued the investigation by maintaining the optimal balance of VPP and ω-VOPO_4_, as obtained in the “a” treatment case.

### Role of Oxygen during the Thermal Treatment

**Figure 2:**
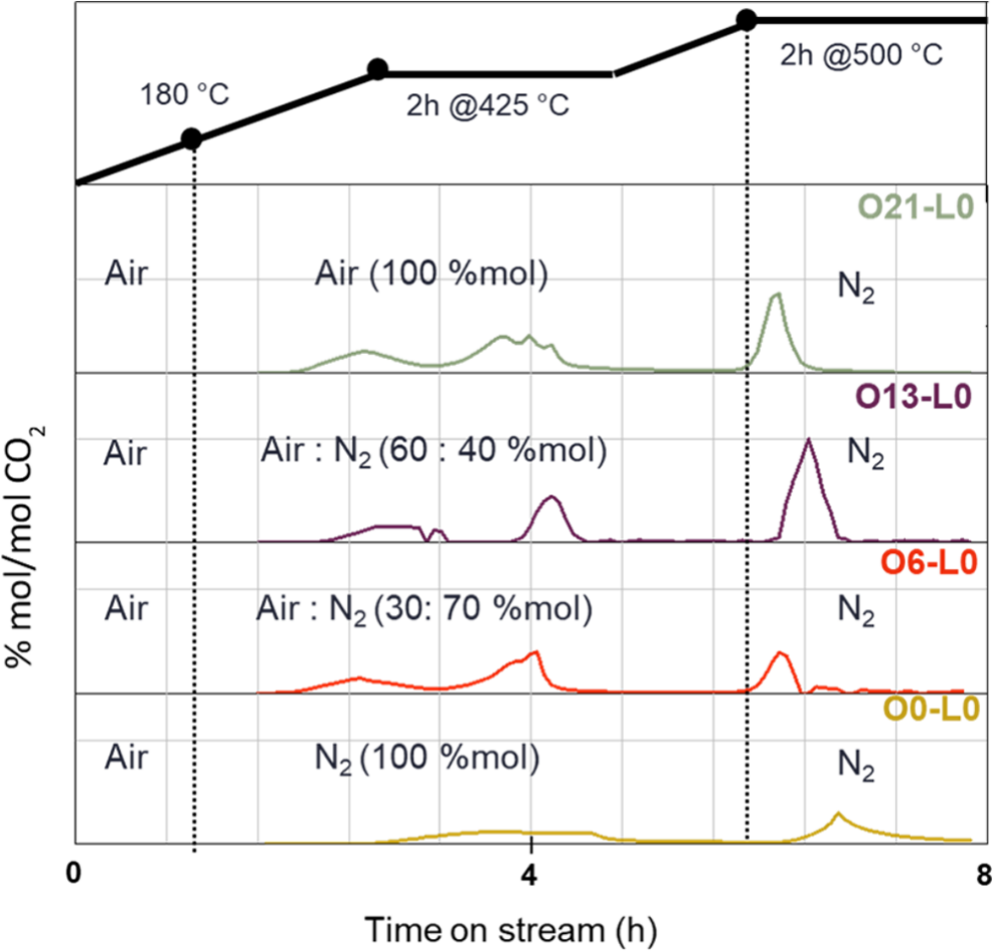
Chromatograms of CO_2_ formation over time-on-stream (h) during the calcination of the sample and temperature (black) for samples O21-L0 (green—O_2_:N_2_:H_2_O 21:79:0), 013-L0 (violet—O_2_:N_2_:H_2_O 13:87:0), 06-L0 (red—O_2_:N_2_:H_2_O 6:94:0), and O0-L0 (yellow—O_2_:N_2_:H_2_O 0:100:0).

Based on the observations from the preliminary tests, further experiments were conducted to correlate the variation in oxygen partial pressure with the structure and reactivity of the catalyst. The samples (all derived from precursor M17) were labeled O21-L0, O13-L0, O6-L0, and O0-L0, corresponding to the molar percentages of oxygen (O) and water (L) used in STEP 2 during calcination (Table 1). During the calcination process, chromatograms were collected using an online micro-GC instrument to monitor the formation of carbon dioxide and water with relative oxygen consumption. Figure 2 shows the formation of CO_2_ over time-on-stream. Considering sample O21-L0, the most significant desorption of water and CO_2_ occurred at 3.5 h time-on-stream, along with the associated oxygen consumption, indicating the combustion of organic residues and the transformation of VHP into VPP. Upon switching the treatment atmosphere to N_2_ and reaching 500 °C, additional CO_2_ formation was detected. This is likely related to the consecutive oxidation processes of partially oxidized organic species formed in previous steps and trapped within the catalyst structure. In the absence of external oxygen, this process should have occurred with both concomitant partial reduction and water retention by the catalyst. Indeed, as shown in the thermal treatment chromatogram of O6-L0 (Figure S5), CO_2_ evolution is not accompanied by water release. Samples O13-L0 and O6-L0 exhibited similar trends even though they were treated with less oxidizing mixtures. For sample O0-L0, calcined in a nonoxidizing atmosphere, significant combustion occurs at 500 °C, with only a limited extent (less than 1% CO_2_ detected) below this temperature.

**Figure 3:**
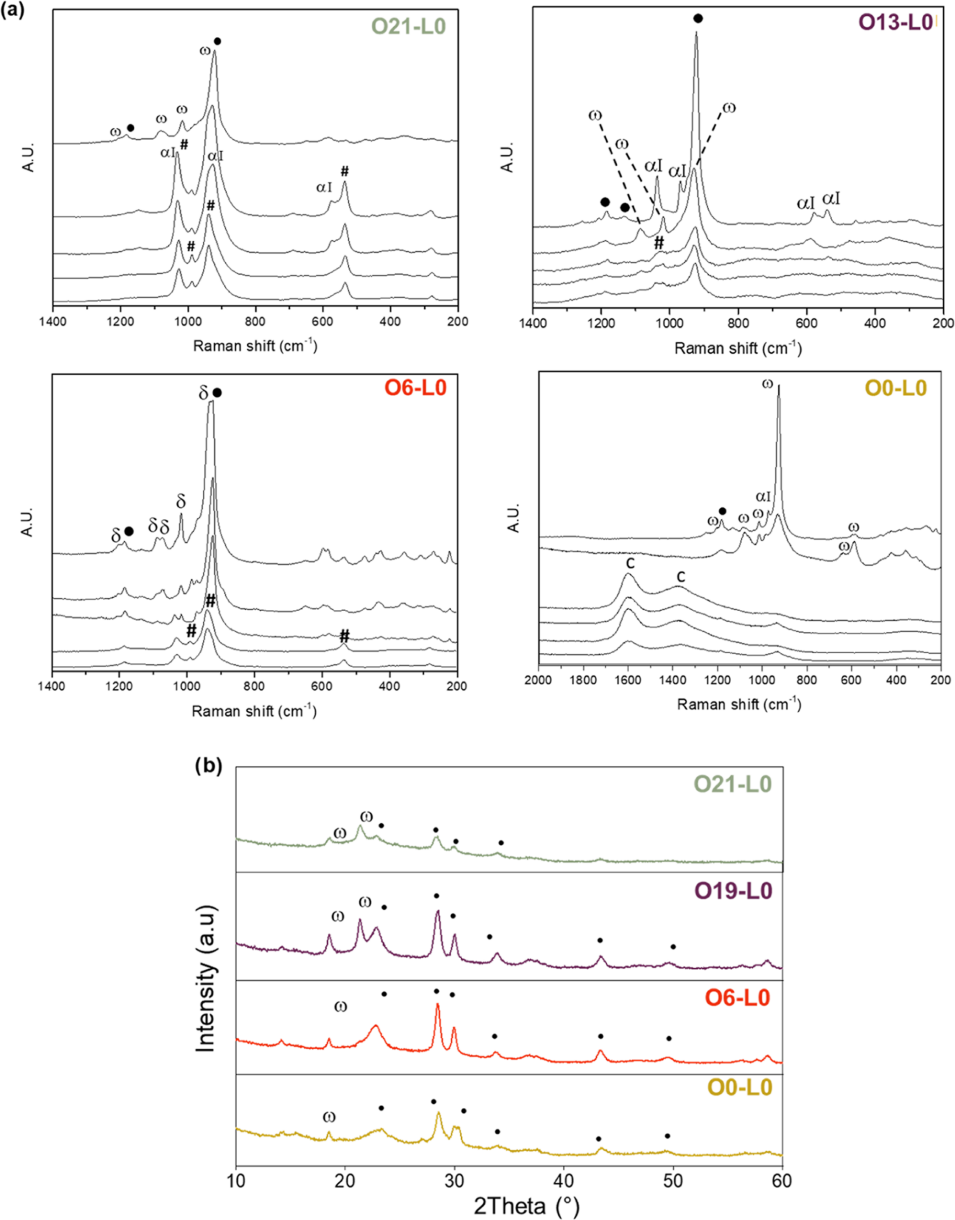
(a) Raman spectra of samples O21-L0 (green—O_2_/N_2_/H_2_O 21:79:0), 013-L0 (violet—O_2_/N_2_/H_2_O 13:87:0), 06-L0 (red—O_2_/N_2_/H_2_O 6:94:0), and O0-L0 (yellow—O_2_/N_2_/H_2_O 0:100:0) after calcination. ω: ω-VOPO_4_; ●: VPP; α_I_: α_I_-VOPO_4_; δ: δ-VOPO_4_; C: carbonaceous species, and #: VOPO_4_·2H_2_O. For each sample, the spectra were collected from different surface spots of possible interest (different shapes, colors, etc.). (b) Diffractograms of the same samples after calcination. ω: ω-VOPO_4_ and ●: VPP.

*Ex situ* Raman analysis (Figure 3a) enabled the detection of the surface composition of the calcined samples (Table S2). O21-L0 mainly contained oxidized vanadium phases (α_I_-VOPO_4_ and VOPO_4_·2H_2_O), with traces of VPP. O13-L0 contains a mix of compounds, including α_I_-VOPO_4_, VPP, and ω-VOPO_4_. O6-L0 showed VPP and δ-VOPO_4_ (1200, 1090, 1075, 1020, and 590 cm^–1^) as the primary phases, with VOPO_4_·2H_2_O. O0-L0 spectra revealed the presence of carbonaceous compounds (bands at 1590 and 1376 cm^–1^) with only traces of oxidized phases and the absence of VPP, indicating that oxygen during the heat treatment is necessary for the transformation. Conversely, the decrease in oxygen content promoted the formation of VPP by reducing the amount of VOPO_4_. X-ray patterns (Figure 3b) showed VPP and ω-VOPO_4_ in all samples, but their proportions varied with the oxygen content used for the thermal treatment. O21-L0 mostly showed oxidized V phosphate with minimal VPP, O13-L0 showed a more defined VPP pattern, and the chromatograms of O6-L0 showed mainly VPP with traces of vanadium orthophosphate. O0-L0 exhibited low crystallinity due to carbonaceous species but with a detectable VPP pattern.

### Reactivity of V/P/O Catalysts

**Figure 4:**
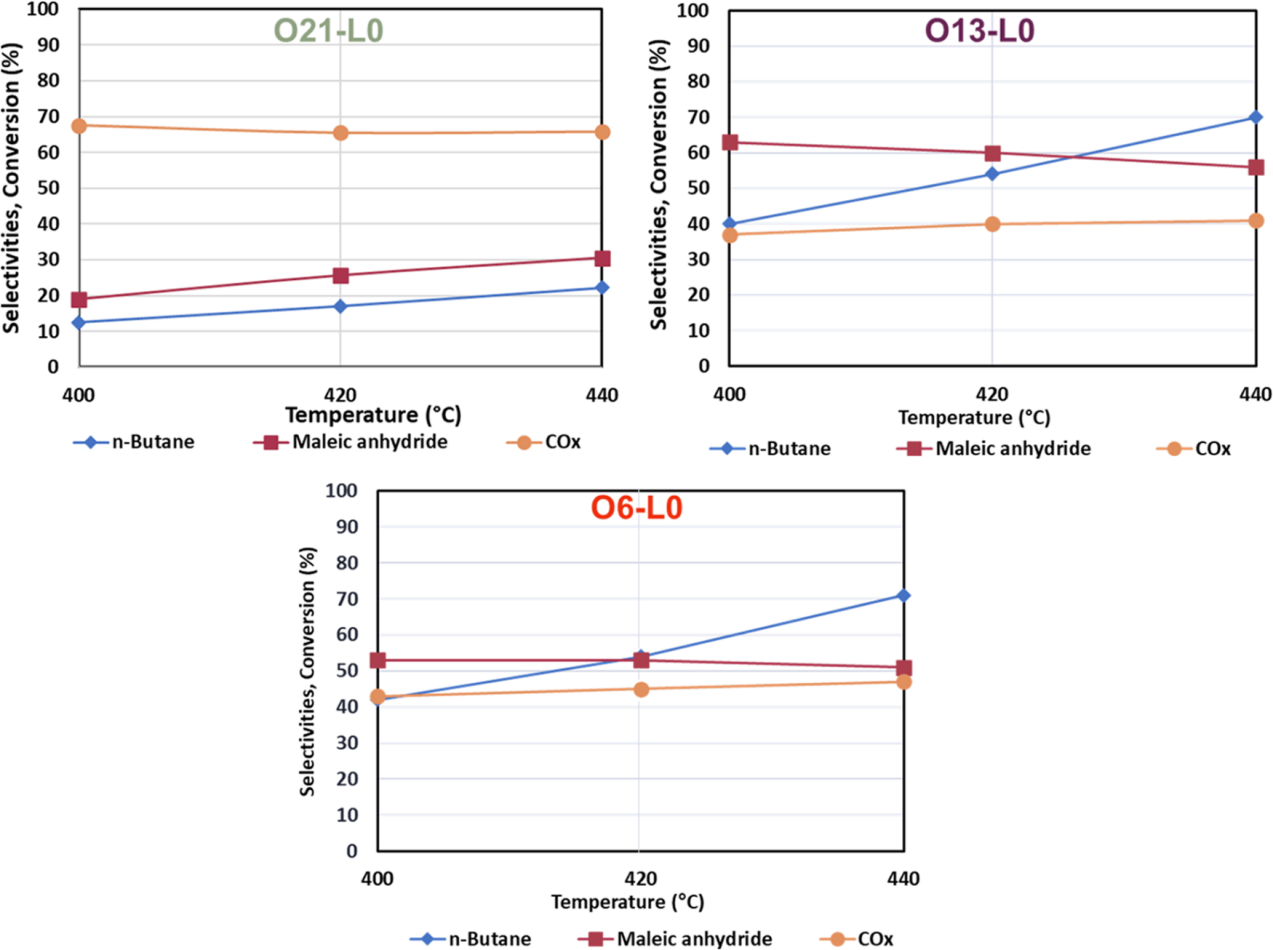
*n*-Butane conversion (blue), MA (purple), and CO*_x_* (orange) selectivity for O21-L0 (green—O_2_/N_2_/H_2_O 21:79:0), O13-L0 (violet—O_2_/N_2_/H_2_O 13:87:0), and 06-L0 (red—O_2_/N_2_/H_2_O 6:94:0) as a function of temperature. Feed composition: 1.7% mol *n*-butane, 17% mol oxygen, remaining inert.

Samples were tested for *n*-butane oxidation at 400, 420, and 440 °C (Figure 4); after a 50 h equilibration step at 400 °C, steady catalytic performance was achieved (not shown). Sample L0-O0 was not tested due to its unsuitable composition. First, a blank test (Figure S6) was performed to test the reactivity of *n*-butane in the gas phase without the catalyst between 360 and 440 °C, thus showing less than 10% conversion due to only CO and CO_2_ formation. This implies that the presence of the V/P/O catalyst is fundamental for MA formation.

O21-L0 exhibited poor performance with low selectivity for MA and high CO_*x*_ selectivity at all temperatures. The MA selectivity and *n*-butane conversion are initially very low at 400 °C, with values of 19 and 13%, respectively. These values increased to 30 and 22%, respectively, as the temperature increased to 440 °C. This poor catalytic activity was attributed to the composition of the catalyst, which contained almost exclusively α_I_-VOPO_4_. Moreover, VOPO_4_·2H_2_O could increase the amount of α_I_-VOPO_4_ present since, under reaction conditions, it could convert into α_I_ if the P/V is sufficiently low.^25^ O13-L0 and O6-L0 showed an improved catalytic performance with *n*-butane conversion of 40% at 400 °C and around 70% at 440 °C, consistent with the fact that they present higher VPP content. O6-L0 can be explained by the presence of δ-VOPO_4_ on its surface without any other unselective compound. Again, XRD characterization of the spent catalyst (Figure S7) confirmed these trends: O21-L0 displayed the presence of α_I_-VOPO_4_ and minimal VPP, and the O13-L0 model showed a sharper VPP pattern and ω-VOPO_4_. O6-L0 exhibited a pattern typical of VPP with traces of ω-VOPO_4_, and the compromise between V^4+^ and V^5+^ phosphates may explain its superior performance.

In summary, it is possible to state that decreasing the oxidizing power of the mixture used for the thermal treatment of the catalyst precursor M17 promotes the formation of VPP. Moreover, the molar percentage of oxygen significantly affects the sample composition and the average V oxidation state (Table S2). As expected, sample O21-L0 exhibits a higher V oxidation state due to calcination in a more oxidizing atmosphere. Conversely, the V oxidation state is lower in the O6-L0 sample, calcined in a poorly oxidizing mixture. The overoxidized sample O21-L0 does not show satisfactory catalytic performance while less oxidizing conditions (O6-L0) yield a favorable balance of V/P/O compounds and improved catalytic activity and selectivity.

### Thermal Treatment in the Presence of Steam

Previous literature studies have highlighted that the presence of water during thermal treatment promotes the formation of the VPP active phase.^21,22,26^ Therefore, several experiments were conducted by varying the % mol of water in the gaseous mixture. This adjustment involved modifying the molar concentrations of oxygen and nitrogen while maintaining the reactor at atmospheric pressure. The catalysts derived from precursor M17 were named O19-L10, O13-L40, and O6-L70, corresponding to 10, 40, and 70 mol % water (L) in the feed, respectively, and compared with sample O21-L0. The volumetric mixtures used for calcination during STEP 2 are listed in Table 1.

**Figure 5:**
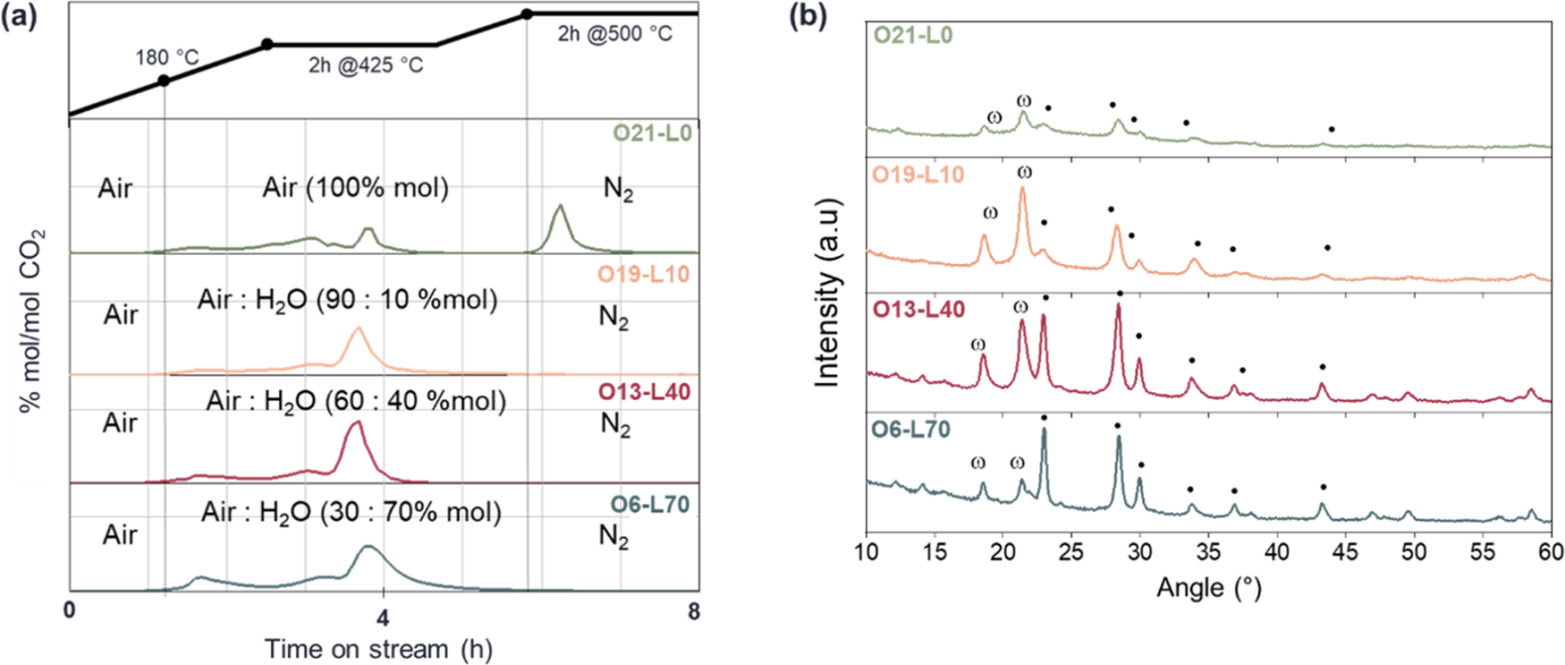
(a) Chromatograms of CO_2_ formation over time-on-stream (h) during calcination of samples O21-L0 (green—N_2_/O_2_/H_2_O 79:21:0), O19-L10 (light orange—N_2_/O_2_/H_2_O 71:19:10), O13-L40 (purple—N_2_/O_2_/H_2_O 47:13:40), and O6-L70 (light blue—N_2_/O_2_/H_2_O 24:6:70) related to the heating ramp; and (b) diffractograms of the same samples after calcination. ω: ω-VOPO_4_ and ●: VPP.

Figure 5a shows the chromatograms illustrating CO_2_ formation as a function of time-on-stream. CO_2_ evolution for samples O19-L10, O13-L40, and O6-L70 is similar, mainly occurring at 425 °C due to the combustion of organic compounds trapped in the catalyst layers during the synthesis. Conversely, O21-L0 exhibited lower CO_2_ evolution in the same temperature range, followed by significant evolution of the same at 500 °C during the isothermal step in N_2_, related to consecutive oxidation of partially oxidized species formed in previous steps and trapped within the catalyst structure. This difference can be attributed to the role of steam in promoting the reformation of organic residues, ensuring their complete combustion below 425 °C.

**Figure 6:**
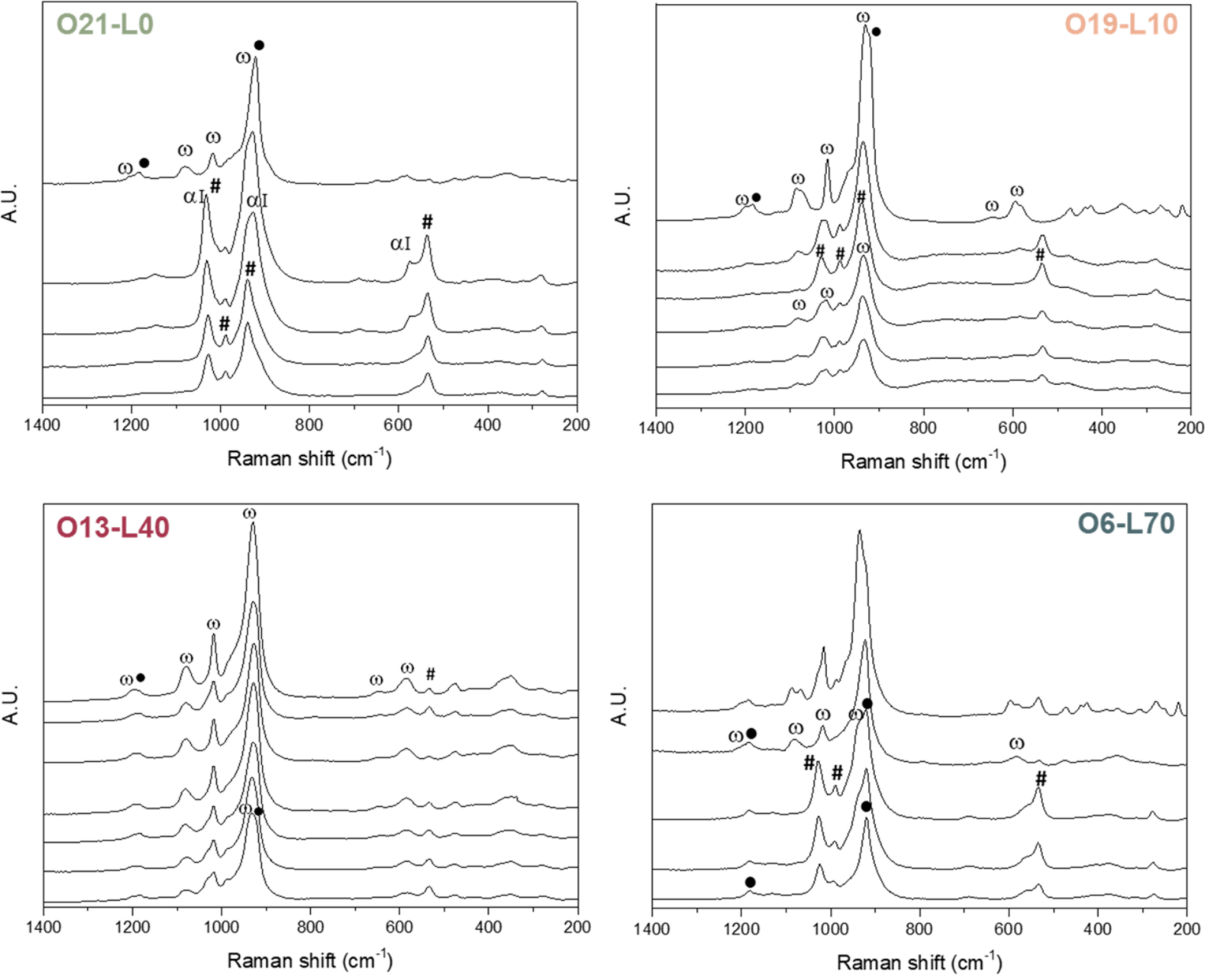
Raman spectra of samples after calcination in different atmospheres: O21-L0 (green–N_2_/O_2_/H_2_O 79:21:0), O19-L10 (light orange—N_2_/O_2_/H_2_O 71:19:10), O13-L40 (purple—N_2_/O_2_/H_2_O 47:13:40), and O6-L70 (light blue—N_2_/O_2_/H_2_O 24:6:70). ω: ω-VOPO_4_; ●: VPP, α_I_: α_I_-VOPO_4_; and #: VOPO_4_·2H_2_O. For each sample, the spectra were collected from different surface spots of possible interest (different shapes, colors, etc.).

The samples were characterized using X-ray diffraction (Figure 5b) and Raman spectroscopy (Figure 6). The specific compounds detected via Raman spectroscopy are listed in Table S2. As a general trend, all the samples calcined with water exhibit ω-VOPO_4_, VOPO_4_·2H_2_O, and VPP in different proportions. O19-L10 displays oxidized V^5+^ phases with VPP present only in traces; O13-L40 and O6-L70, calcined with higher % mol of water, presented more varied distributions of phases. The copresence of ω-VOPO_4_ and VPP as the main phases was suggested by the presence of broad bands at 1190 and 920–930 cm^–1^ due to band overlap. The main difference relies on the higher amount of VPP detected in O6-L70, suggesting a greater V^4+^ concentration, especially on the catalyst surface. This condition could lead to highly active and selective catalysts for the target reaction.

The XRD pattern (Figure 5b) confirmed these findings but highlighted the differences in crystallinity (Figure S8). O21-L0 exhibited very low crystallinity compared to all the other samples calcined with steam. Increasing the water partial pressure led to higher overall sample crystallinity, and focusing on reflexes at 23 and 28° 2θ, it is possible to see an increase of crystallinity for VPP at the expense of ω-VOPO_4_ (18 and 21° 2θ), consistent with Raman spectroscopy observations.

The surface area determination (Table S2) showed no direct relationship between the surface area and crystallinity based on the water content in the mixture, but the overall V oxidation state aligned with the XRD findings. Increasing the amount of water in the calcination mixture and, consequently, reducing the oxygen molar concentration decreased the overall V oxidation state.

To summarize, water plays a crucial role in the thermal treatment of VHP to produce the final V/P/O catalyst. Water could facilitate structural reorganization by accelerating combustion reactions at lower temperatures, allowing the catalyst more time to recrystallize. Increasing the water partial pressure during calcination promotes the formation of the VPP active phase, reduces the amount of oxidized phase (VOPO_4_), and leads to a well-designed catalyst for the target reaction.

### Reactivity of the V/P/O Catalysts after the Thermal Treatment in the Presence of Steam

**Figure 7:**
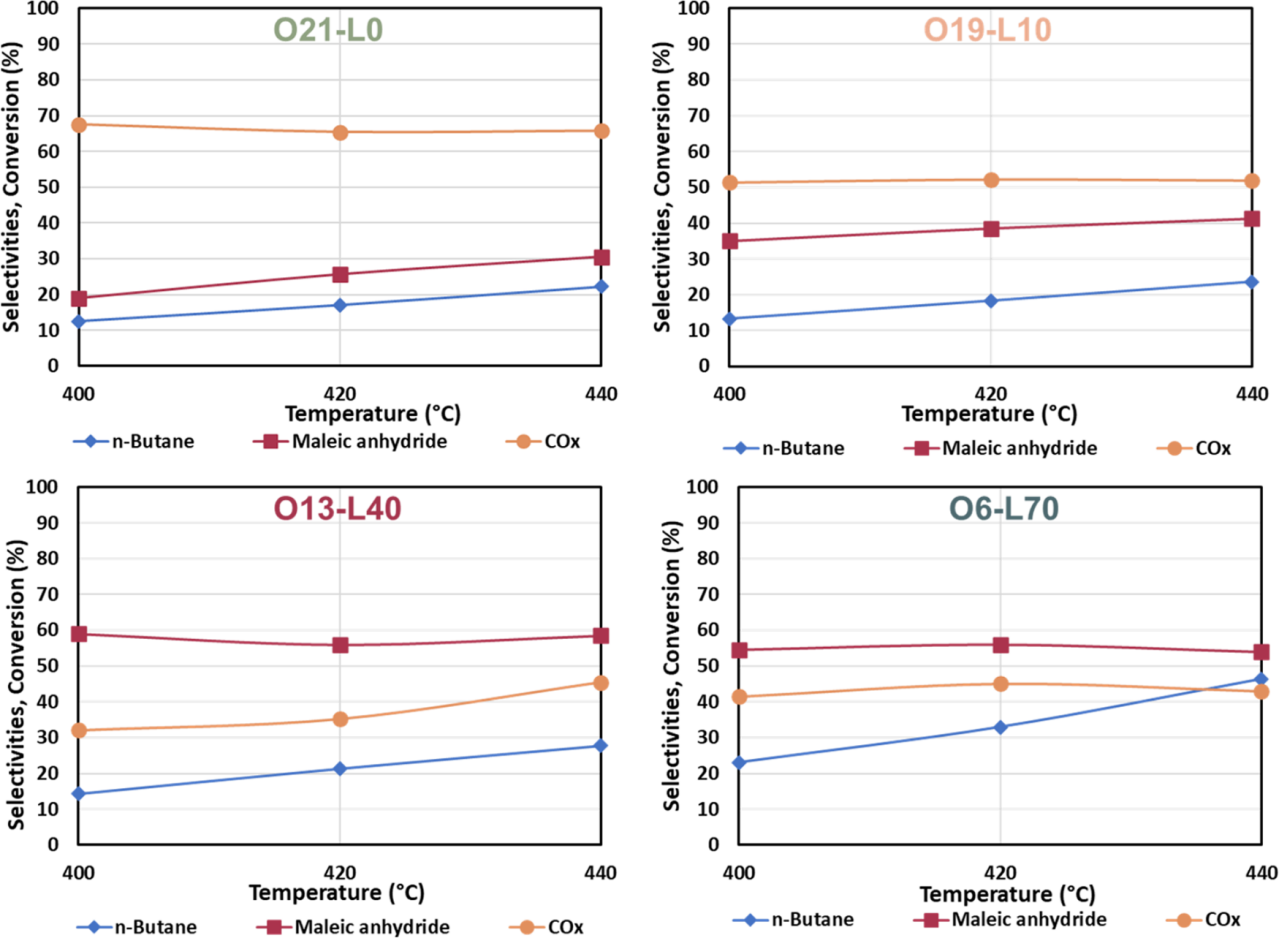
*n*-Butane conversion (blue), MA (purple), and CO_*x*_ (orange) of O21-L0 (green—N_2_/O_2_/H_2_O 79:21:0), O19-L10 (light orange—N_2_/O_2_/H_2_O 71:19:10), O13-L40 (purple—N_2_/O_2_/H_2_O 47:13:40), and O6-L70 (light blue—N_2_/O_2_/H_2_O 24:6:70) as a function of temperature. Feed composition: 1.7% mol *n*-butane, 17% mol oxygen, remaining inert; *W*/*F* = 1.33 g·s·mL^–1^.

Figure 7 shows the catalytic performance of the catalysts. The catalysts were initially equilibrated for 50 h at 400 °C, and then reactivity experiments were carried out at 400, 420, and 440 °C. The O21-L0 complex showed poor reactivity, as previously discussed. O19-L10 demonstrated a slightly higher activity, with MA selectivity reaching 35% at 400 °C and increasing to 41% at higher temperatures; CO_*x*_ selectivity remained high and constant at 52%. The differences in selectivity and activity can be explained by the presence of ω-VOPO_4_, which can convert into selective δ-VOPO_4_ at the reaction temperature,^26^ as well as the limited amount of VPP. Increasing the water partial pressure during thermal treatment (O13-L40) led to better MA selectivity, close to 57% at all temperatures. The CO_*x*_ selectivity increased from 32% at 400 °C to 45% at 440 °C. The O6-L70 complex showed the best performance, with conversion rates ranging from 22% at 400 °C to 46% at 440 °C, along with a stable MA selectivity of around 55%. This can be attributed to the better balance between ω-VOPO_4_ and VPP in the fresh catalyst.

**Figure 8:**
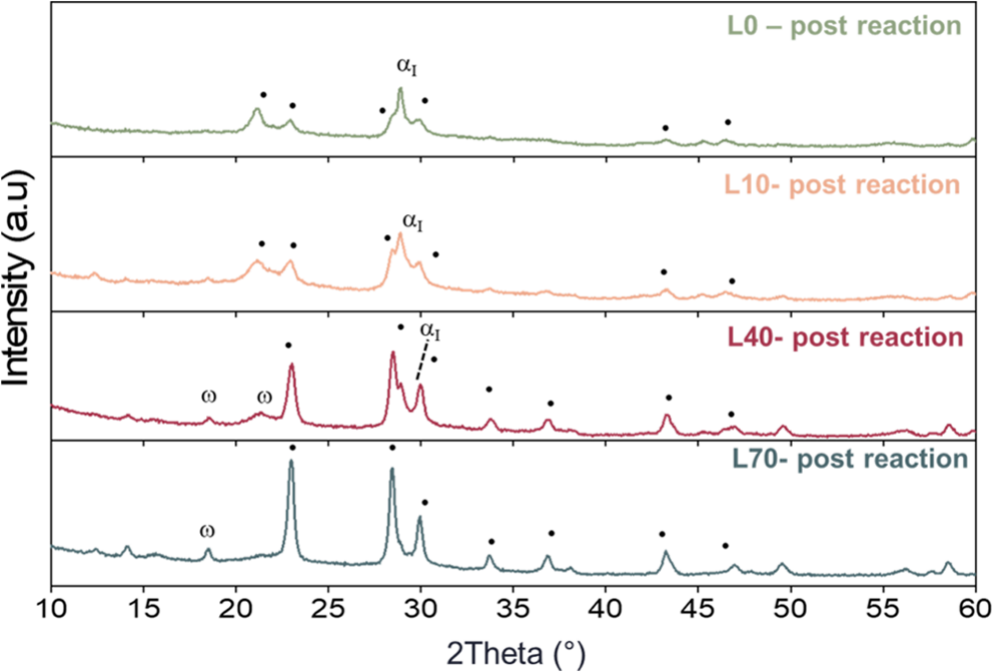
XRD pattern after the reaction of O21-L0 (green—N_2_/O_2_/H_2_O 79:21:0), O19-L10 (light orange—N_2_/O_2_/H_2_O 71:19:10), O13-L40 (purple—N_2_/O_2_/H_2_O 47:13:40), and O6-L70 (light blue—N_2_/O_2_/H_2_O 24:6:70). ω: ω-VOPO_4_; α_I_: α_I_-VOPO_4_; and ●: VPP.

The characterization of the spent catalyst provided further insights. XRD patterns (Figure 8) confirmed the role of the water partial pressure; a higher water partial pressure reduced the formation of oxidized compounds (α_I_-VOPO_4_ and ω-VOPO_4_) in favor of VPP. O21-L0 and O19-L10, which still showed the presence of oxidized compounds in both fresh and spent catalysts, demonstrated poor performance. O13-L40, with sharper VPP peaks but still dominant VOPO_4_ compounds, was less active. O6-L70, with VPP as the main phase and traces of ω-VOPO_4_, exhibited the best performance.

Raman characterization (Figure S9) supported these findings. O21-L0 and O19-L10 primarily exhibited α_1_-VOPO_4_, while the cations in O13-L40 also showed δ-VOPO_4_ and VPP. O6-L70 displayed a favorable distribution of phases, mainly VPP and δ-VOPO_4_ with traces of α_I_-VOPO_4_, justifying its stable selectivity.

In conclusion, increasing the water partial pressure during thermal treatment produces a stable catalyst that does not undergo any further phase transformations under the reaction conditions. To further validate this statement, Figure S10 presents the catalytic performance of O6-L70 as a function of time-on-stream at the three investigated temperatures. As expected, the catalyst maintained stable MA and CO_2_ selectivity for over 12 h.

### Optimization of the Gas Mixture for Thermal Treatment

The experimental results for O6-L70 and O6-L0 reveal some notable observations. Both samples exhibited high MA selectivity (∼70%), as shown in Figures 4 and 7. However, their activities differed significantly, with O6-L0 demonstrating markedly higher activity than O6-L70. This similar selectivity can be attributed to the identical partial pressure of oxygen used during the thermal treatment (Table 1), which influenced the composition, as previously mentioned. Both samples displayed the same bulk composition (VPP and selective ω-VOPO_4_), as confirmed by XRD characterization (Figures 3b and 5b) and share the same average V oxidation state of 4.21 (Table S2).

The difference in activity can be explained by XRD analysis. For V/P/O catalysts, an ordered stacking of the (200) planes results in a lower intensity ratio (*I*_042_/*I*_200_), leading to higher MA yields.^26^ Additionally, the structural disorder of catalysts depends on the calcination atmosphere. In the presence of water, the disorder along the (200) planes decreases, which is indicated by the “aspect ratio” that considers the full width at half-maximum of the two relevant peaks at 2θ = 28° (042) and 2θ = 22.5° (200) (FWHM_042_/FWHM_200_). This ratio provides insights into the exposure of the (100) plane, which exposes a large fraction of active sites.^25^ A broadening of the (200) line indicates higher catalytic activity without affecting the nature of the active sites and the overall selectivity.^14,27,28^ Moreover, the partial pressure of water accelerates the removal of alcohol molecules trapped between the layers, influencing the structural recrystallization and broadening of the (200) line. O6-L0 exhibits a higher temperature for organic residue combustion than O6-L70, resulting in a less crystalline catalyst with higher disorder along the (200) plane. O6-L70, instead, shows a more crystalline and less disordered structure.

**Figure 9:**
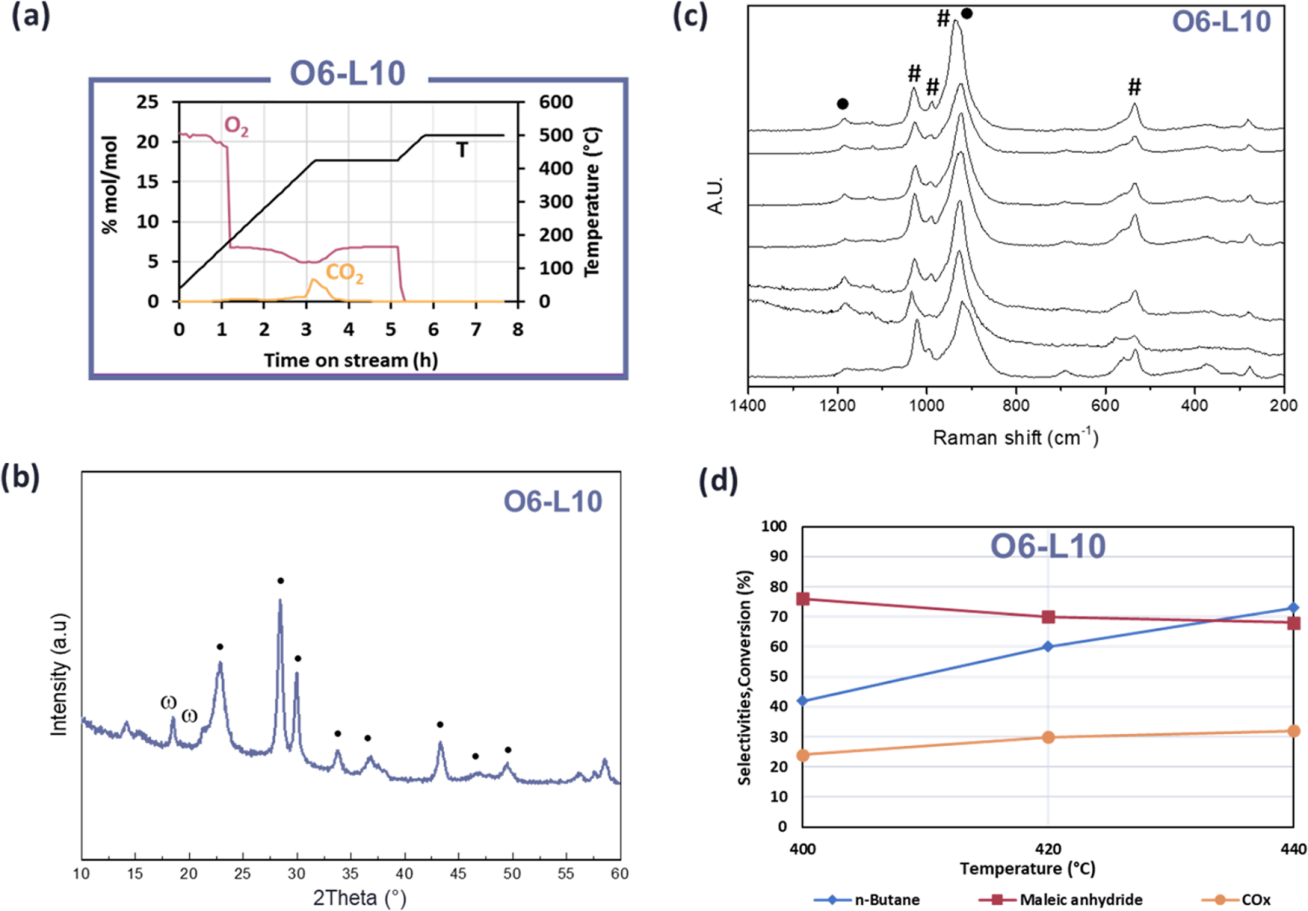
(a) Calcination trends of O_2_ (purple), CO_2_ (orange), and temperature (black) for samples O6-L10 (violet—O_2_/N_2_/H_2_O 6:84:10); (b, c) Raman and X-ray characterization after calcination. ω: ω-VOPO_4_; ●: VPP, and #: VOPO_4_·2H_2_O. Various spectra were collected from different surface spots of possible interest (different shapes, colors, etc.); and (d) *n*-butane conversion (blue), MA (purple), and CO_*x*_ (orange). Feed composition: 1.7% mol *n*-butane, 17% mol oxygen, remaining inert; W/*F* = 1.33 g·s·mL^–1^.

These findings suggest the need for a compromise between the oxygen and water contents co-fed during calcination to achieve optimal performance. Therefore, a new sample (O6-L10) was prepared with a minimal amount of oxygen and water during thermal treatment. Details regarding the calcination, characterization, and catalytic activity of this sample are shown in Figure 9.

Figure 9a shows that 10% water resulted in marked CO_2_ formation during calcination between 350 and 425 °C, with no further combustion of organic residues, consistent with the observations from other samples calcined in the presence of water. The XRD pattern (Figure 9b and S11) indicates modest crystallinity, as in the O6-L0 case, suggesting a similar (200) plane exposure. The composition (VPP and ω-VOPO_4_) mirrors that of O6-L0 and O6-L70, as expected, given the same oxygen content used during calcination. For the same reason, the average V oxidation state is the same as that for the other two samples (4.20). The surface area is 11 m^2^/g, which is consistent with the values previously obtained.

The Raman spectra (Figure 9c) reveal that the peaks of O6-L10 are quite homogeneous, with both VOPO_4_·2H_2_O and VPP in every recorded spectrum. Focusing on the main signal between 920 and 930 cm^–1^, it appears broadened and centered at 925 cm^–1^, whereas in O6-L0 and O6-L70, it is centered at 927 and 919 cm^–1^, respectively. This difference can highlight the existence of various VPP polytypes (structures differing in the symmetric relationship between the adjacent dimeric vanadium polyhedra), as already hypothesized by different authors.^10,13,29−32^ It has been demonstrated that the preparation and calcination conditions, such as the presence of water, influence the formation of different structures. However, it is still not clear which specific conditions favor one structure over another. The hypothesis of the coexistence of different VPP polytypes in O6-L10, as inferred from the FWHM ratios of the (042) and (200) planes, is enforced by the presence of intermediate intensity and width ratio values when compared to those of O6-L0 and O6-L70.

Catalytic testing (Figure 9d) shows superior performance: at 400 °C, it achieved 75% MA selectivity and 41% butane conversion, and the latter increased to 70% at 440 °C while maintaining 70% MA selectivity. This suggests that the gas mixture composition used for thermal treatment was optimized. Moreover, the possible coexistence of different polytypes in the sample could explain the high activity and MA selectivity. Indeed, the O6-L10 sample probably compromises the (200) plane exposure and lowers the FWHM ratio.^26^ Characterization of the spent O6-L10 sample displays minimal changes in the XRD pattern compared with the fresh one, with some α_I_-VOPO_4_ spots (Figure S12). These findings highlight again the crucial role of water during calcination. An optimal water and oxygen balance enhances catalyst crystallinity, morphology, and composition, thereby improving activity and selectivity.

### Effect of the Precursor Type

As mentioned above, the two industrial precursors exhibit different properties (i.e. P/V ratio, amount of carbon and vanadium average oxidation state), potentially leading to different catalytic performances. Several calcination tests were performed with precursor M16 to evaluate differences and similarities with M17. The new thermal treatments produced samples O19-L10_M16_, O13-L40_M16_, O6-L0_M16_, O6-L10_M16_, and O6-L70_M16_, which were obtained using the same heating ramp and atmospheres as for the M17-derived samples (Table 1). Their characterization is reported in Figures S12–S14 and summarized in Table 4.

**Table 4:**
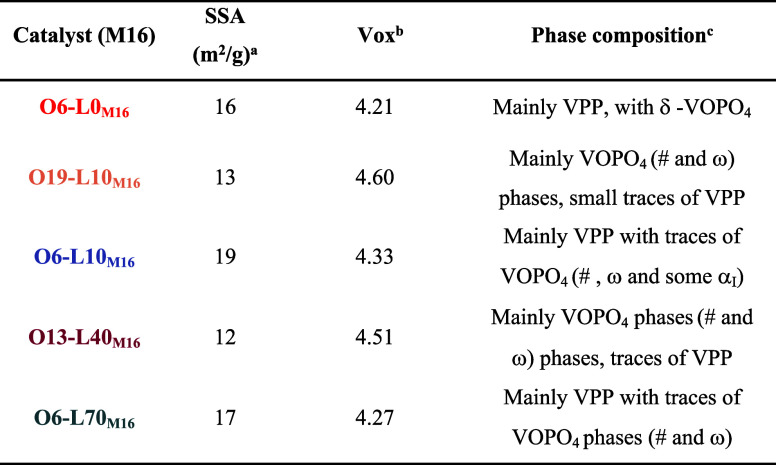
Characterization of Samples O6-L0_M16_, O19-L10_M16_, O6-L10_M16_, O13-L40_M16_, and O6-L70_M16_ after Calcination

a Overall oxidation state of vanadium.

b Determined by titration and specific surface area.

c Determined by nitrogen physisorption with an associated error of 3% and phase composition with Raman spectroscopy.

The plots recorded during calcination (Figure S14) show that the samples from M16 present the same behavior as those obtained from M17: water accelerates the combustion of the carbon residues, resulting in a lower temperature for the major CO_2_ release for samples calcined in the presence of water (O19-L10_M16_, O13-L40_M16_, O6-L10_M16_, and O6-L70_M16_) compared to that of O6-L0_M16_. Interestingly, all the samples present lower temperatures for organic residue combustion than the M17-derived catalysts, likely due to the M16 lower carbon content (Table 2), which may facilitate combustion, thus allowing a longer time for sample crystallization. For this reason, M16-derived catalysts are expected to be more crystalline than M17-derived ones.

Concerning the XRD characterization (Figure S15), all of the considerations are the same as for the M17-derived samples: all patterns display VPP and ω-VOPO_4_ as the main components, in a relative amount affected by the oxygen content during thermal treatment. However, unlike the M17-derived catalysts, samples calcined with a higher oxygen content showed the presence of the dihydrate phase; the latter can convert into selective δ-VOPO_4_. An increase in the oxygen content enhanced the crystallinity of the sample, as was also observed for the M17 catalysts. Raman spectra (Figure S16) did not show any particular difference between the two series of catalysts, except for a more consistent presence of the dihydrate VOPO_4_ in the M16 samples, as highlighted by the corresponding XRD patterns. Notably, under the same calcination atmosphere, the M16-derived catalysts present a higher vanadium average V oxidation state (Table 4), which is likely attributable to the lower carbon content (C_in_) of the precursor, allowing gaseous oxygen to oxidize vanadium after completing the combustion of organic species. In addition, M16 presents a higher amount of V^5+^ species in the precursor (0.04%, compared to 0.006% in M17), contributing to the higher V oxidation state in the M16-derived catalysts. The SSA is generally higher in M16-derived samples since the starting precursor displayed a higher SSA. Moreover, the lower C content could guarantee a less hindered catalyst surface resulting in a higher area.

**Figure 10:**
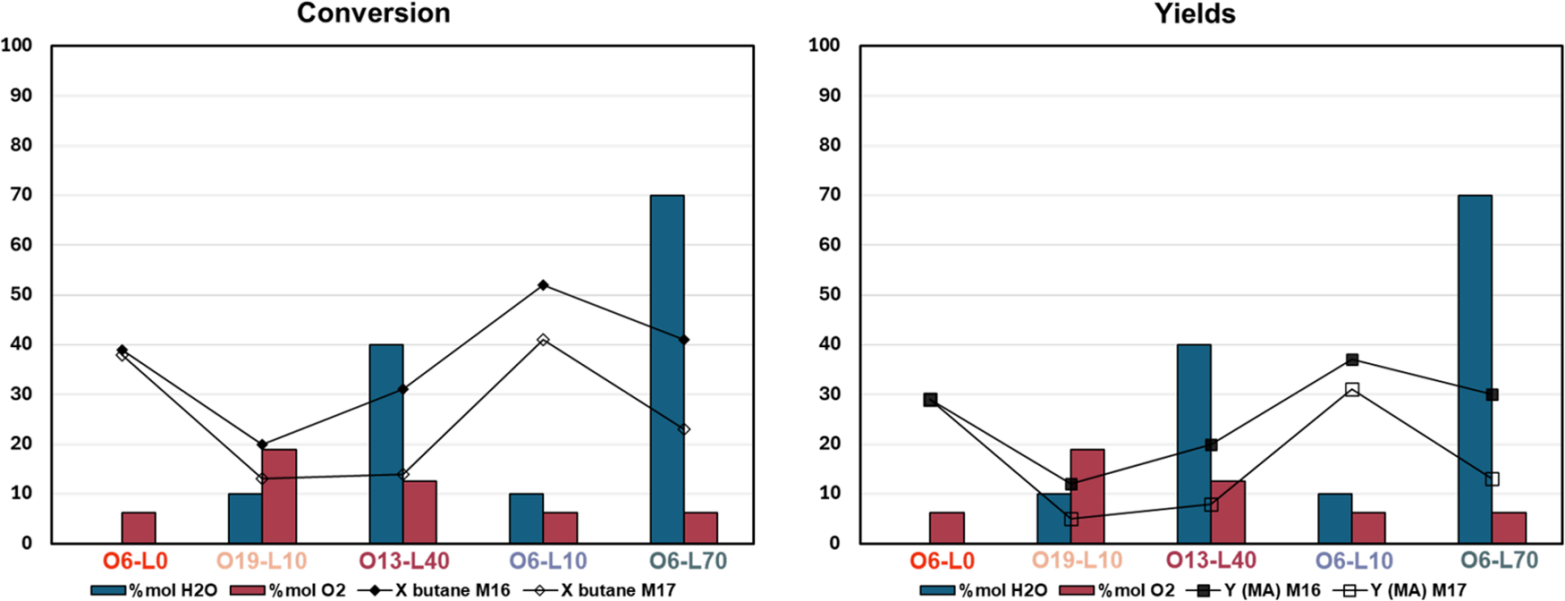
*n*-Butane conversion (left, ⧫ ◊) and MA yield (right, ■ □) as a function of O_2_ (red bars) and H_2_O (blue bars) molar content in the calcination atmosphere for M16 (full symbol) and M17 (open symbol). The data are ordered with decreasing amounts of oxygen from left to right. Reaction conditions: *T* = 400 °C; *W*/*F* = 1.33 g·s/mL, *n*-butane/O_2_/inert 1.7:17: remaining (% mol).

The catalysts were tested under the usual reaction conditions; Figure 10 shows the yields and *n*-butane conversions obtained compared with the results obtained with M17 catalysts.

In general, M16-derived catalysts present better performance than M17, probably because of the higher P/V atomic ratio. It is well known from the literature that a lower P content may give rise to a less selective catalyst because of the higher formation of α_I_-VOPO_4_. On the other hand, higher P/V atomic ratios in M16 promote the conversion of VOPO_4_·2H_2_O into selective δ-VOPO_4_. This hypothesis was confirmed by looking at the characterization of the spent catalysts: all samples displayed the δ-phase in the Raman spectra and some in the bulk phase (as visible from the O6-L70_M16_ characterization in Figure S17). Moreover, as previously mentioned, the lower C content in the M16 precursor allows for faster combustion of organics during thermal treatment, giving the structure more time to reorganize during precursor transformation.

To further assess the effect of the calcination atmosphere on the morphology and P/V ratio of the catalyst, a series of selected catalysts, obtained after the thermal treatment of the M17 precursor in the presence of different mol % of either O_2_ or water, were further characterized by SEM-EDX analyses and compared with the best-performing catalyst obtained with M16 (O6-L10_M16_, Figure S13). Interestingly, the M17 samples calcined in the absence of steam (“L0”) maintained a regular and spherical morphology, which is derived from the industrial spray-drying step used in the synthesis of the VHP precursors, with only small differences in terms of rugosity of the surface while moving from higher to lower O_2_ mol % (namely, O21-L0 and O6-L0). However, by increasing both the %mol steam and by decreasing the O_2_ %mol in the feed, important differences in terms of morphology can be underlined (see M17-derived samples, namely O21-L0, O19-L10, O6-L10, and O6-L70 in Figure S13). In particular, the spheres start to deform due to the growth of crystalline, lamellar, and flower-like structures over the surface of the spheres of the pristine material (particularly evident for the O6-L70 sample), also leading to the coalescence of different spheres. On the other hand, by comparing the two different precursors treated under the same conditions, namely O6-L10_M17_ and O6-L10_M16_, a higher tendency of M16 to undergo these surficial transformations, with the formation of a crystalline lamellar structure and higher surface defects compared to the M17 analogues, can be observed. Notably, the P/V ratios of the surficial crystals of samples O6-L10_M16_ and O6-L70 _M17_ are between 1.22 and 1.24, which are significantly higher than the P/V values obtained for the residual spheres, which are close to 1.16 (see Table S3). All these aspects, i.e., different morphologies with higher rugosity, the presence of defects, and higher P/V ratios of the crystals, will probably contribute to the enhanced catalytic activity observed for these samples. On the contrary, the very high amount of water fed during the calcination step in the case of O6-L70_M17_ led to extensive degradation and coalescence of the spheres, leading to a concomitant decrease of the specific surface area, thus explaining the maximum catalytic performance of the samples obtained with a moderate amount of water (i.e., 10 mol %, as shown in Figure 10).

Finally, the performances of the two catalysts obtained from industrial precursors M16 and M17 through thermal treatment under optimized conditions (O6-L10_M16_, O6-L10_M17_) are compared with those of other VPO-based catalysts reported in recent literature (Table S4). The performance in terms of *n*-butane conversion and selectivity to maleic anhydride is comparable to the literature data, confirming that optimization of the calcination atmosphere, which is gaining traction in the scientific community, can also have an immediate impact on improving the performance of industrially established catalysts.

### Surface Features of Calcined Catalysts

The acidity of VPP catalysts has been explored in several works.^21,33,34^ Zhang et al.^21^ reported that an increased content of steam enhanced the B/L ratio in VPP due to the partial hydrolysis of V–O–P bonds on the VPP surface. Moreover, steam increases the P/V atomic ratio on the catalyst surface compared to the bulk ratio; this ratio was reported not to change significantly before and after the reaction, indicating the stabilizing effect induced by steam treatment. Steam also affects the distribution of V species between the bulk and the surface; thus, the existence of stable VOPO_4_ compounds was hypothesized. In our previous work,^25^ we found by XPS that in two used “equilibrated” catalysts having different P/V ratios (but both prepared in the absence of steam during thermal treatment), the P/V ratio, close to 1.8, was still higher than the corresponding bulk values but similar in the two cases. This occurs because even though the VPP catalyst holds a surface P-enrichment, during the reaction, part of the excess P is lost; this explains why in industrial operation, it is necessary to feed volatile P-containing compounds in order to keep a P-enriched surface.^25^

We characterized the selected samples by DRIFT spectroscopy after the adsorption of pyridine (Figure S18). In particular, three M17 samples calcined either in the absence or in the presence of the minimum amount of steam (O6L0_M17_, O6L10_M17_, and O19-L10_M17_) showed negligible Brønsted acidity, with a B/L ratio of about 0.09 only in the case of the O19-L10_M17_ sample, which suggests the role of both the higher P/V ratio (as high as 1.21) and the presence of steam in affecting the final Brønsted surface acidity. On the other hand, a further increase of the amount of steam up to 70 mol % in the calcination environment clearly promoted the formation of Brønsted acid sites in agreement with Zhang et al.^21^ (i.e., see sample O6-L70_M17_, Figure S18, B/L ratio ≈2). Interestingly, this increased Brønsted acidity is also correlated with a shift to lower wavenumbers of all the main bands attributed to Lewis acid sites, suggesting a weaker interaction with the probe molecule. This weaker acidity might partially be responsible for the decline of both butane conversion and MA yield shown in Figure 10, for the samples calcined with the higher mol % of steam. Finally, the samples calcined under the best conditions with the two different precursors, O6L10_M17_ and O6L10_M16_, showed slightly different B/L ratios of 0.04 and 0.16, respectively. These results are consistent with the higher P/V ratio of the M16-derived material (P/V of 1.31 compared to 1.18 of M17), which favors the formation of Brønsted acid sites when calcined in the presence of a suitable amount of steam.

## Conclusions

Herein, a systematic investigation of the influence of different thermal treatment conditions (i.e., gas-phase composition during the calcination step) on the final properties and catalytic activities of different VPO materials is reported. In particular, two industrial VHP precursors for the fluid-bed reactor, delivered by Polynt S.p.A, were used for this study, differing in P/V ratio, residual C content, and vanadium oxidation states; these properties are affected by the preparation procedures used at the industrial level. The in situ monitoring of the compounds released during the thermal treatment allowed us to understand that steam plays an important role in the removal of the organic residues still present in the precursor, thus facilitating the crystallization of VPP. Therefore, the presence of steam during activation is fundamental with catalyst precursors showing a greater amount of residual organic compounds retained in the crystalline structure. Moreover, experimental evidence was obtained, highlighting the role of steam not only in affecting the nature of the VPP polytype formed in the thermal process but also in determining the nature of the acidic sites over the catalyst surface. Interestingly, increasing the amount of steam in the feed to 70 mol % leads to the promotion of Brønsted acid sites via V–O–P bond hydrolysis, which is detrimental to the catalytic performance. Notably, using a calcination mixture composed of 6 and 10% mol of oxygen and water, respectively, diluted in nitrogen, it is possible to obtain outstanding performance (40% conversion of *n*-butane and 73% selectivity at 400 °C). Moreover, optimization (i.e., minimization) of the amount of steam required offers several advantages from an industrial perspective, such as reducing the energy costs of the vaporization process. Finally, the role of intrinsic properties of the precursors in the catalytic performance was investigated: under the same calcination conditions, the M16-derived catalyst presented enhanced reactivity. The first reason can be related to the higher P/V ratio that guarantees the formation of dihydrated VOPO_4_, which can be converted into active δ-VOPO_4_. The present study allowed us to understand that the thermal treatment has to be carefully designed as a function of the characteristics of the catalyst precursors, thus enhancing both the selectivity and activity of the final catalytic material.

## Data Availability

The data underlying this study are available in the published article and its Supporting Information.

## Supporting Information Available

Additional details for the experimental section; flow sheets of the equipment; and characterization of the samples (PDF)
