# Supplementary material for: New Insights into the Key Role of Thermal Treatment in V/P/O Catalysts for the Selective Oxidation of n-Butane to Maleic Anhydride
Source: ACS Omega. 2025 Feb 11;10(7):7452–65. doi: 10.1021/acsomega.5c00501 (PMC11865973; doi:10.1021/acsomega.5c00501)
Supplement: Supplementary file 1 — ao5c00501_si_001.pdf [file ao5c00501_si_001.pdf]

New insights into the key role of the thermal treatment of V/P/O catalysts for the selective oxidation of *n*-butane to maleic anhydride

Ludovica Conte,[a,b] Laura Setti,[a] Giacomo Luzzati,[a,b] Tommaso Tabanelli,[a,b] Laura Fratalocchi,[c] Lorenzo Grazia,[c] Silvia Luciani,[c] Silvia Bordoni,[a] Carlotta Cortelli,[c] Fabrizio Cavani[a,b]*

[a] Dipartimento di Chimica Industriale "Toso Montanari", Università di Bologna, viale Risorgimento 4, Bologna, 40136, Italy;
[b] Center for Chemical Catalysis - C^3, Alma Mater Studiorum Università di Bologna, viale Risorgimento 4, Bologna, 40136, Italy
[c] Polynt SpA, Via E. Fermi 51, 24020 Scanzorosciate (BG), Italy
*tommaso.tabanelli@unibo.it

Electronic Supporting Information (ESI)

Summary

ESI contains 17 Figures:

- Figure S1. Scheme of the bench scale plant used for the thermal treatment.
- Figure S2. Scheme of lab-scale plant for the selective oxidation of n-butane to MA.
- Figure S3. XRD pattern of precursors M16-M17.
- Figure S4. XRD analysis (left) and Raman spectra (right) for samples obtained calcinating M17 precursor with varying atmospheres.
- Figure S5. Chromatograms of CO_2 and water evolution, oxygen consumption, and temperature over time on stream registered during calcination of sample 06-L0.
- Figure S6. n-butane conversion as a function of the reaction temperature in a reactor filled with steatite.
- Figure S7. XRD pattern of samples O21-L0, O13-L0, O6-L0, and O0-L0 after reaction.
- Figure S8. XRD pattern of samples O21-L0, O19-L10, O13-L40, and O6-L70 after calcination, detail for crystallinity.
- Figure S9. Raman spectra of samples O21-L0, O19-L10, O13-L40 after the reaction.
- Figure S10. Selectivities and conversion of catalyst O6-L70 over the time on stream at T=400,420 and 440 °C.
- Figure S11. XRD pattern after calcination of samples O6-L0, O6-L10, and O6-L70, detail for crystallinity.
- Figure S12. XRD pattern and Raman characterization of sample O6-L10 after reaction.
- Figure S13. SEM characterization of samples O6-L0, O19-L10, O6-L10, O6-L70, O21-L0 and sample O6-$L10_{M16}$.
- Figure S14. CO_2 evolution and oxygen consumption during calcination of samples O19-$L10_{M16}$, O13-$L40_{M16}$, O6-$L0_{M16}$, O6-$L10_{M16}$, O6-$L70_{M16}$.

- Figure S15. XRD pattern after calcination of samples O19-L10$_{M16}$, O13-L40$_{M16}$, O6-L0$_{M16}$, O6-L10$_{M16}$, and O6-L70$_{M16}$.
- Figure S16. Raman spectra after calcination of samples O19-L10$_{M16}$, O13-L40$_{M16}$, O6-L0$_{M16}$, O6-L10$_{M16}$, O6-L70$_{M16}$.
- Figure S17. XRD analysis (left) and Raman spectra (right) of sample O6-L70$_{M16}$ after reaction.

4 Tables:

- Table S1. Raman bands of different Vanadium/Phosphorous phases.
- Table S2. Characterization of all the samples cited in this work after calcination (vanadium oxidation state, SSA, and phase composition).
- Table S3. P/V atomic ratio obtained by EDX analysis of samples O6-L10$_{M16}$, O19-L10$_{M17}$, O6-L70$_{M17}$, O6-L0$_{M17}$, O21-L0$_{M17}$, and O19-L0$_{M17}$ after calcination.
- Table S4. Comparison of the catalytic performance of O6-L10$_{M16}$ O19-L10$_{M17}$ with those reported in the literature.

1 Procedure:

- P1. Procedure for chemical analysis of the samples.

P1. Procedure for chemical analysis of the samples

The chemical analysis of samples was performed as follows. The equilibrated sample was dissolved in concentrated fuming sulfuric acid. Then its V content was determined by titration of V^V with a Mohr salt solution (FeII), and V^{IV} was determined by titration with a $KMnO_4$ 0.1 N solution. The Vanadium oxidation state was obtained from the total vanadium content (renamed Vtot) and, later, the amount of V^{5+}:

$$Vox = 4 + \frac{\%\, V^{5+}}{\%\, V_{tot}}$$

Phosphorus was determined gravimetrically as quinoline molybdophosphate.

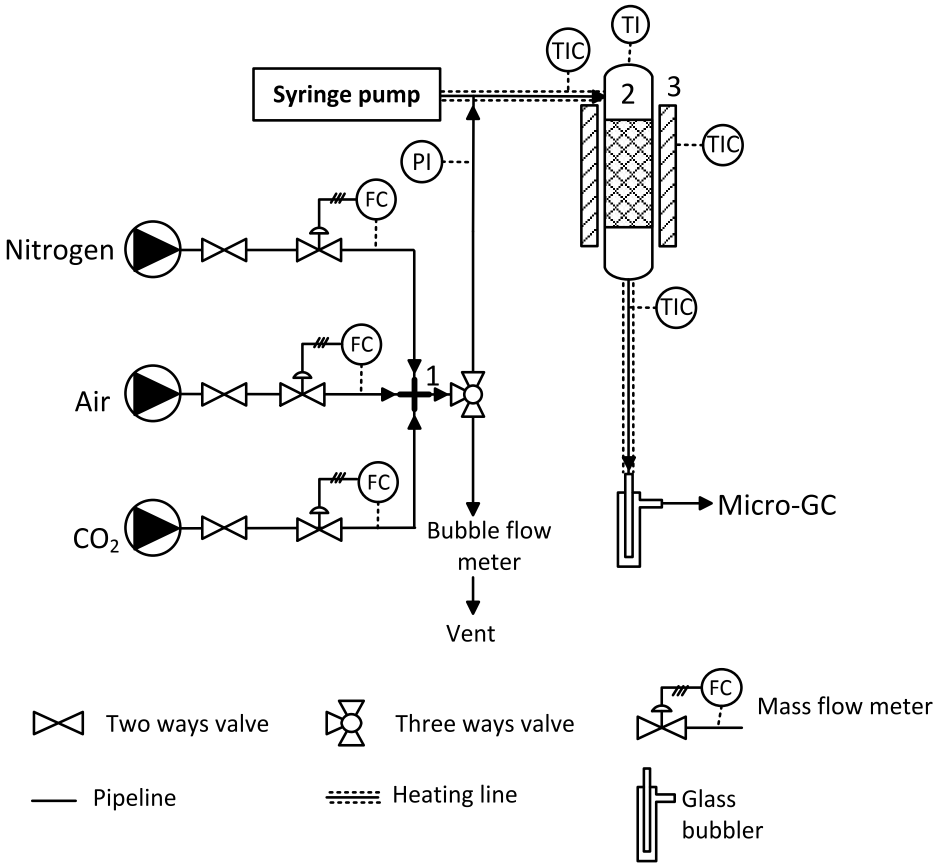

Figure S1. Bench scale plant used for the thermal treatment.

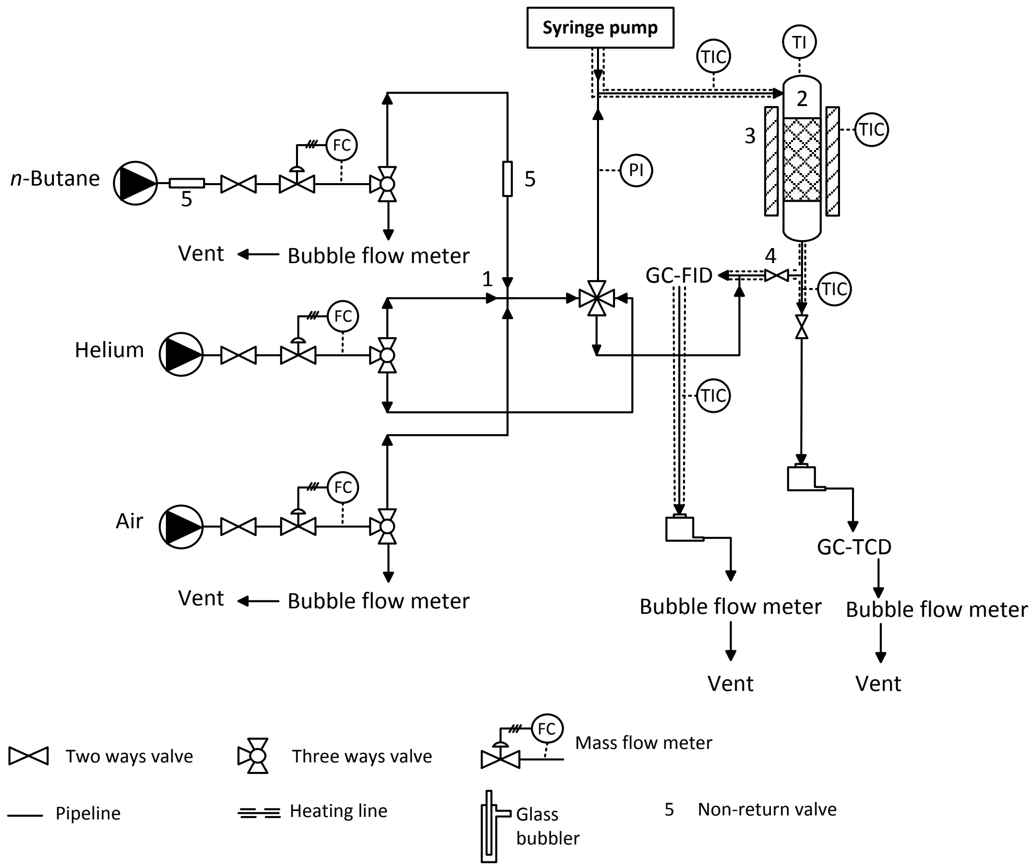

Figure S2. Lab-scale plant for the selective oxidation of *n*-butane to MA.

Table S1. Raman bands of different Vanadium/Phosphorous phases.

V/P/O	cm^{-1}			
	**1200-1100**	**1100-1000**	**1000-900**	**900-400**
$(VO)_2P_2O_7$	1185, 1135		930, 920	
$(VO)HPO_4*0.5H_2O$	1155, 1100		985	342
$VOPO_4*2H_2O$		1039	988, 952	542
α_I-$VOPO_4$		1032	928	579, 541
α_{II}-$VOPO_4$		1091	993, 979, 945	433, 399
δ-$VOPO_4$	1200	1090, 1075, 1020	936	590
β-$VOPO_4$		1075	997, 986	892, 435
ω-$VOPO_4$	1188	1084, 1016	932	650, 589

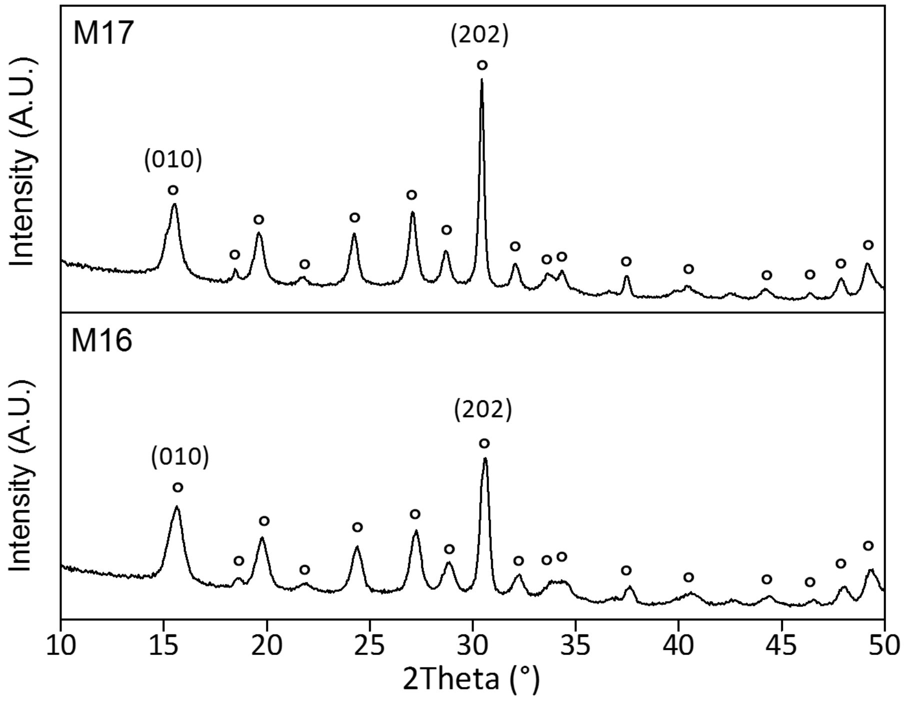

Figure S3. XRD pattern of precursors: top M17, bottom M16. Symbol: ° = crystalline VHP.

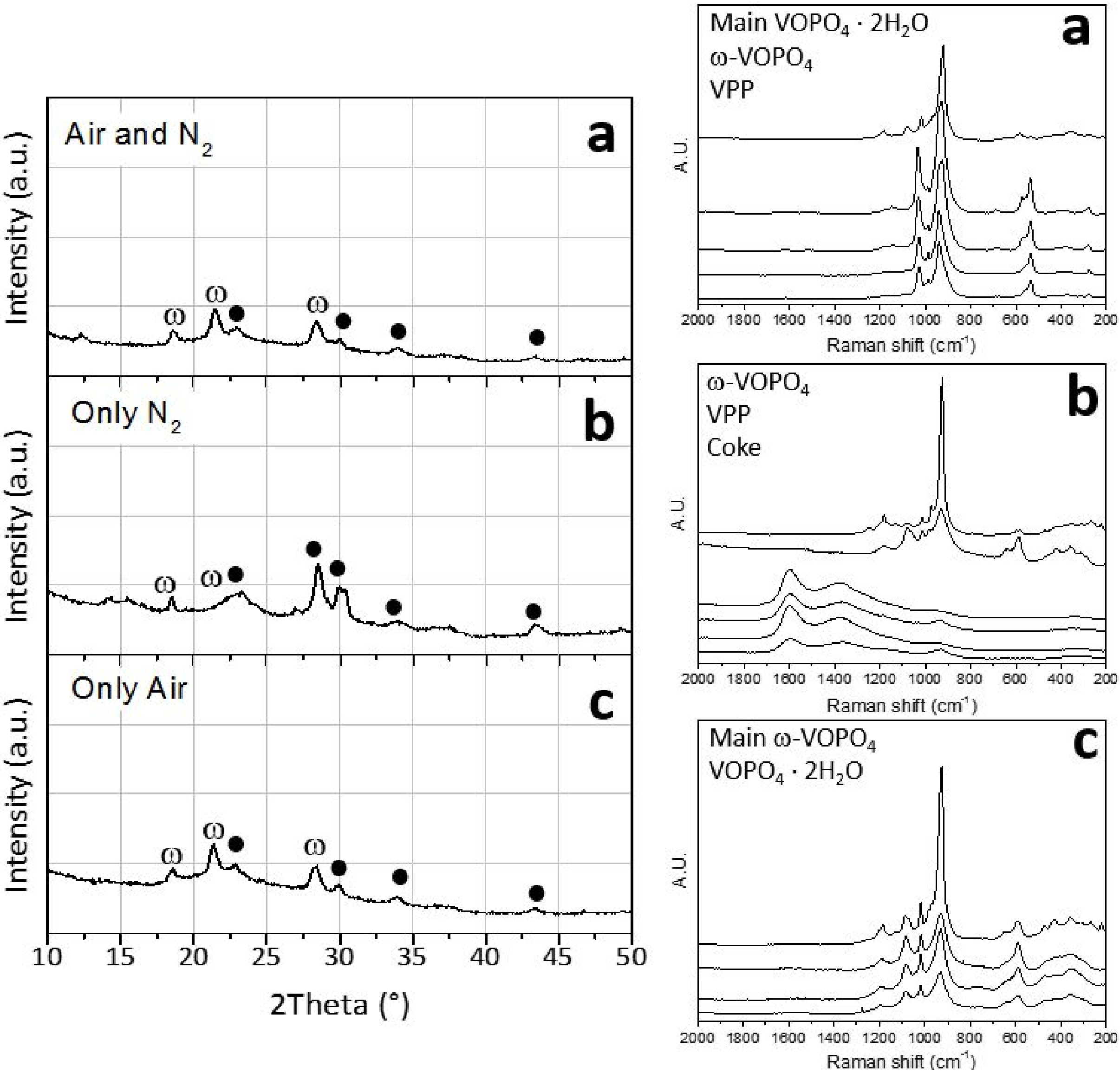

Figure S4. XRD analysis (left) and Raman spectra (right) for samples obtained calcinating M17 precursor with air/nitrogen (a), only nitrogen (b), and only air (c) as a carrier gas for the calcination. Symbols: ω = ω-$VOPO_4$; ● = VPP. For each sample, various Raman spectra were collected from different surface spots of possible interest (different shapes, colors, etc.).

Table S2. Characterization of samples O21-L0, work after calcination. Vanadium's overall oxidation state[a] is determined by titration; specific surface area[b] is determined with nitrogen physisorption with an associated error of 3%; and phase composition[c] with Raman spectroscopy.

Cat.	Vox[a]	SSA (m^2/g)[b]	Phase composition[c]
**O21-L0**	4.52	9	Mainly $VOPO_4$ (α_I, ω and di-hydrate) phases, traces of VPP
**O13-L0**	4.47	12	Varied distribution of $VOPO_4$ (α_I, ω) but richer in VPP
**O6-L0**	4.21	14	Mainly VPP with δ-$VOPO_4$ and traces of dihydrate phase.
**O0-L0**	-	-	Rich in carbonaceous compounds, traces of VPP and $VOPO_4$ (α_I and ω)
**O19-L10**	4.53	12	Mainly $VOPO_4$ (α_I and ω) phases, traces of VPP, and di-hydrate phase
**O13-L40**	4.41	11	Mainly ω -$VOPO_4$ with the consistent presence of VPP; traces of di-hydrate phase
**O6-L70**	4.21	7	Mainly VPP with the presence of ω -$VOPO_4$
**O6-L10**	4.19	11	Mainly VPP with traces of dihydrate phase.
**O6-L0$_{M16}$**	4.27	16	Mainly VPP with traces of δ -$VOPO_4$ phase.
**O19-L10$_{M16}$**	4.60	9	Mainly ω–$VOPO_4$, traces of VPP, and di-hydrate phase
**O13-L40$_{M16}$**	4.41	11	Mainly di-hydrate phase, traces of VPP and α_I-$VOPO_4$
**O6-L70$_{M16}$**	4.27	17	Mainly VPP with traces of dihydrate phase.
**O6-L10$_{M16}$**	4.31	19	Mainly VPP with traces of ω–$VOPO_4$ phase.

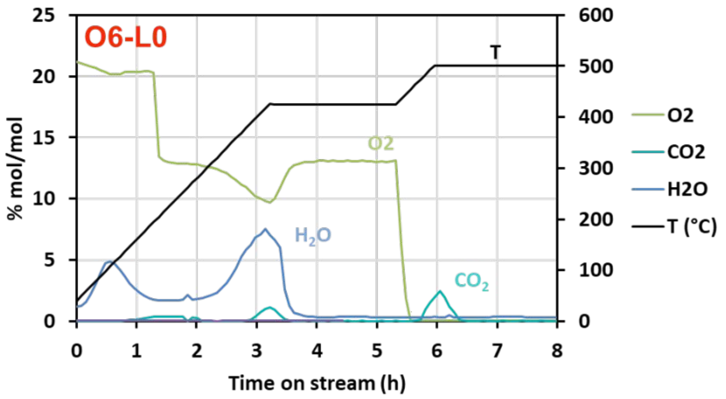

Figure S5. Chromatograms of CO_2 and water evolution (blue and light blue), oxygen consumption (green), and temperature (black) over time on stream registered during calcination of sample 06-L0.

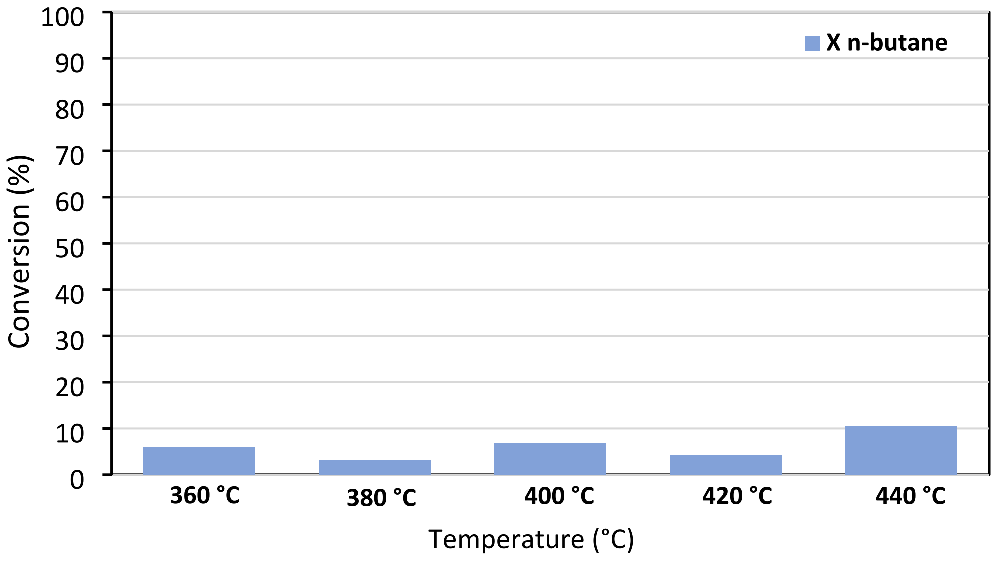

Figure S6. *n*-butane conversion as a function of the reaction temperature in a reactor filled with steatite (light blue bars). Feed composition: 1.7: 17 % mol of n-butane: air, remain inert; W/F: 1.33 g · s · mL^{-1}.

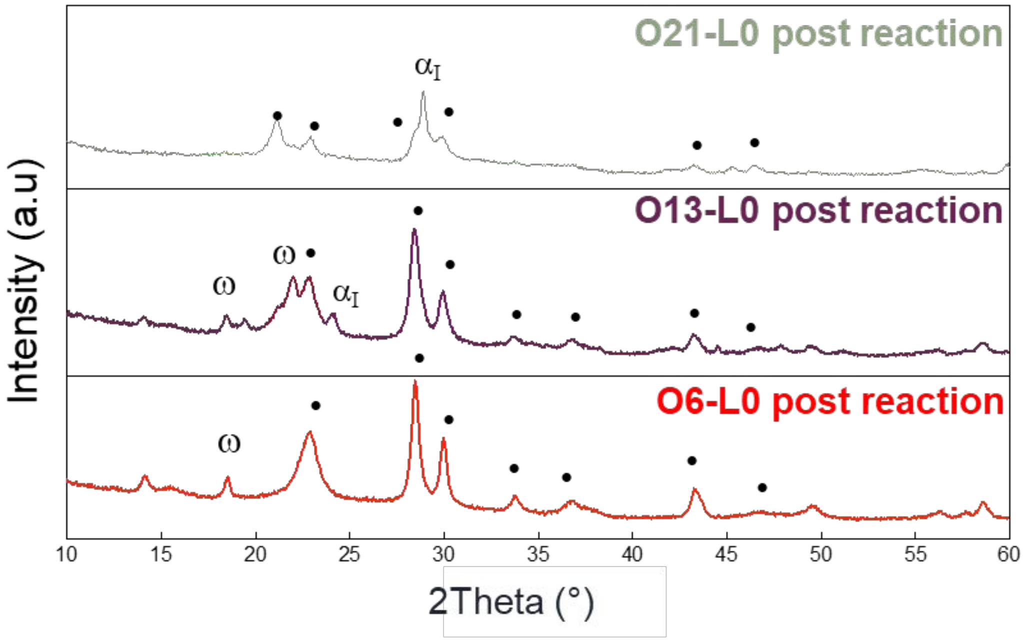

Figure S7. XRD pattern of samples after reaction O21-L0 (green- 21:79:0 O_2:N_2:H_2O); O13-L0 (violet- 13:87:0 O_2:N_2:H_2O; O6-L0 (red- 6:94:0 O_2:N_2:H_2O); O0-L0 (yellow- 0:100:0). Symbols: ω = ω-$VOPO_4$; ● = VPP; α_I = α_I-$VOPO_4$.

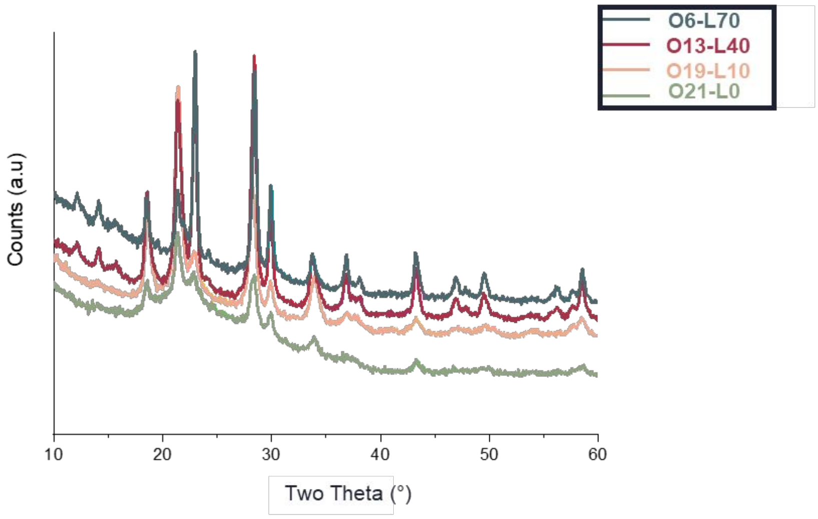

Figure S8. XRD pattern of samples after calcination in different atmospheres: O21-L0 (green-N_2:O_2:H_2O 79:21:0), O19-L10 (light orange- N_2:O_2:H_2O 71:19:10), O13-L40 (purple - N_2:O_2:H_2O 47:13:40), and O6-L70 (light blue- N_2:O_2:H_2O 24:6:70), detail for crystallinity.

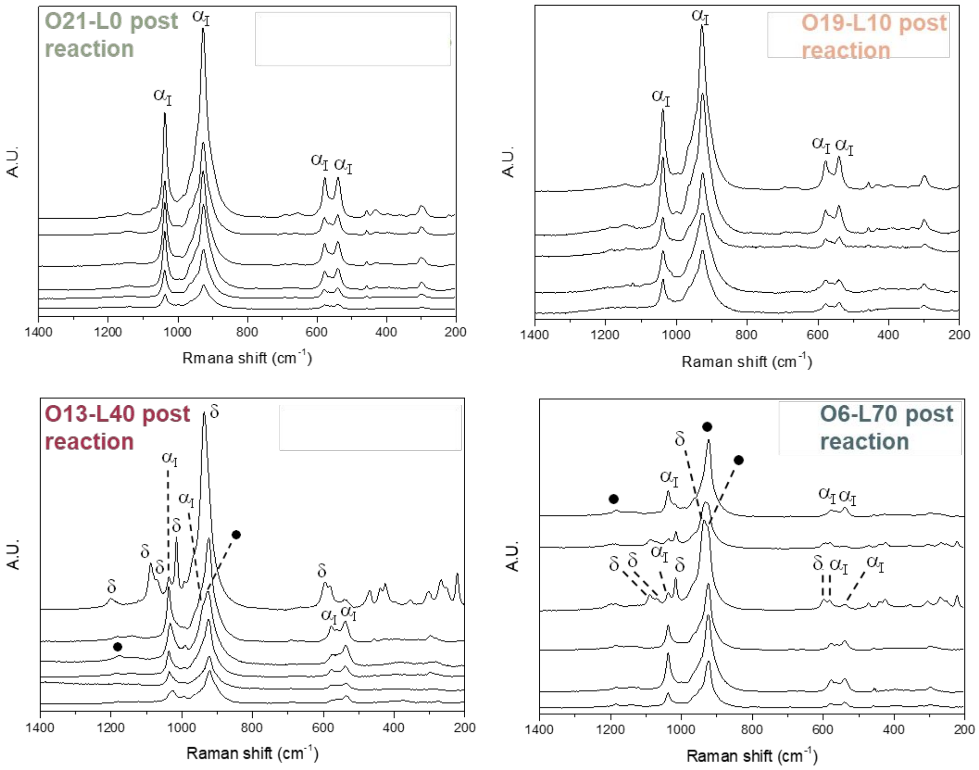

Figure S9. Raman spectra of samples O21-L0 (green-N_2:O_2:H_2O 79:21:0), O19-L10 (light orange- N_2:O_2:H_2O 71:19:10), O13-L40 (purple - N_2:O_2:H_2O 47:13:40), and O6-L70 (light blue- N_2:O_2:H_2O 24:6:70) after the reaction. Symbols: δ= δ-$VOPO_4$; α_I = α_I-$VOPO_4$; ● = VPP. For each sample, various spectra were collected from different surface spots of possible interest (different shapes, colors, etc.).

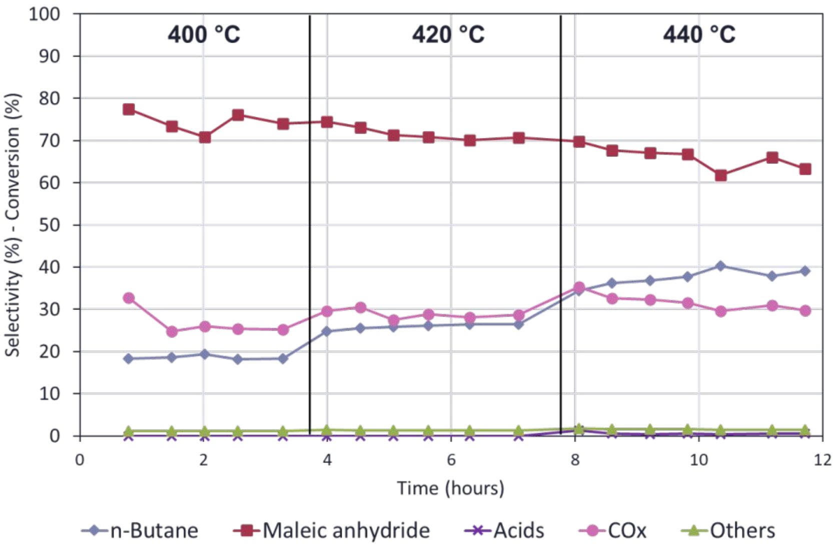

Figure S10. Selectivities (%; red – maleic anhydride, pink – COx, green – Others, violet – Acids) and conversion (%; blue – nButane) of catalyst O6-L70 over the time on stream (h) at T=400,420 and 440 °C. Feed composition: 1.7% mol n-butane, 17%mol oxygen, remain inert, W/F = 1.33 g·s·mL^{-1}.

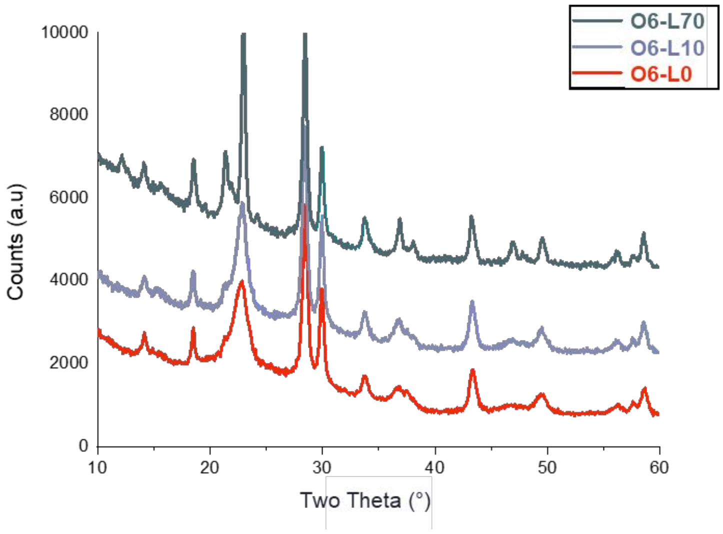

Figure S11. XRD pattern of samples after calcination of samples O6-L0 (red- N_2:O_2:H_2O 94:6:0), O6-L10 (violet- N_2:O_2:H_2O 84:6:10), and O6-L70 (light blue- N_2:O_2:H_2O 24:6:70), detail for crystallinity.

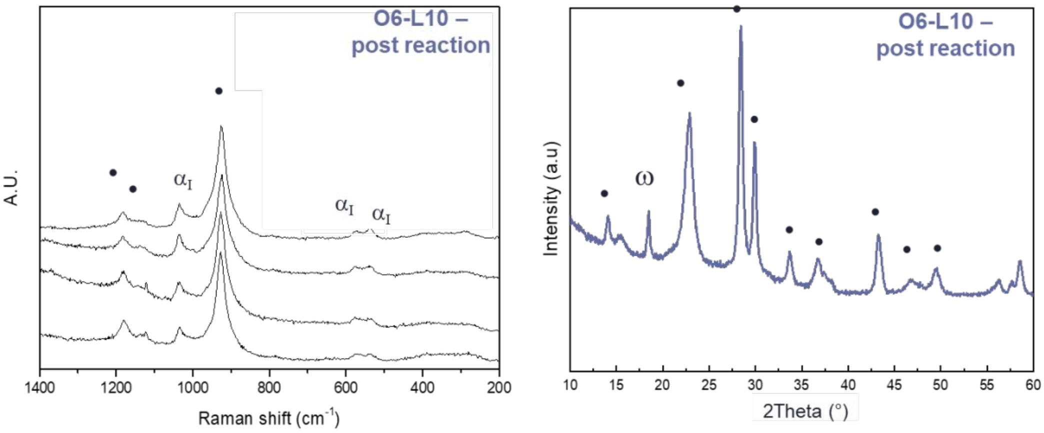

Figure S12. XRD pattern (left) and Raman spectra (right) of sample O6-L10 (violet - N_2:O_2:H_2O 84:6:10) after reaction. Symbols: α_I = α_I-$VOPO_4$; ● = VPP. Various Raman spectra were collected from different surface spots of possible interest (different shapes, colors, etc.).

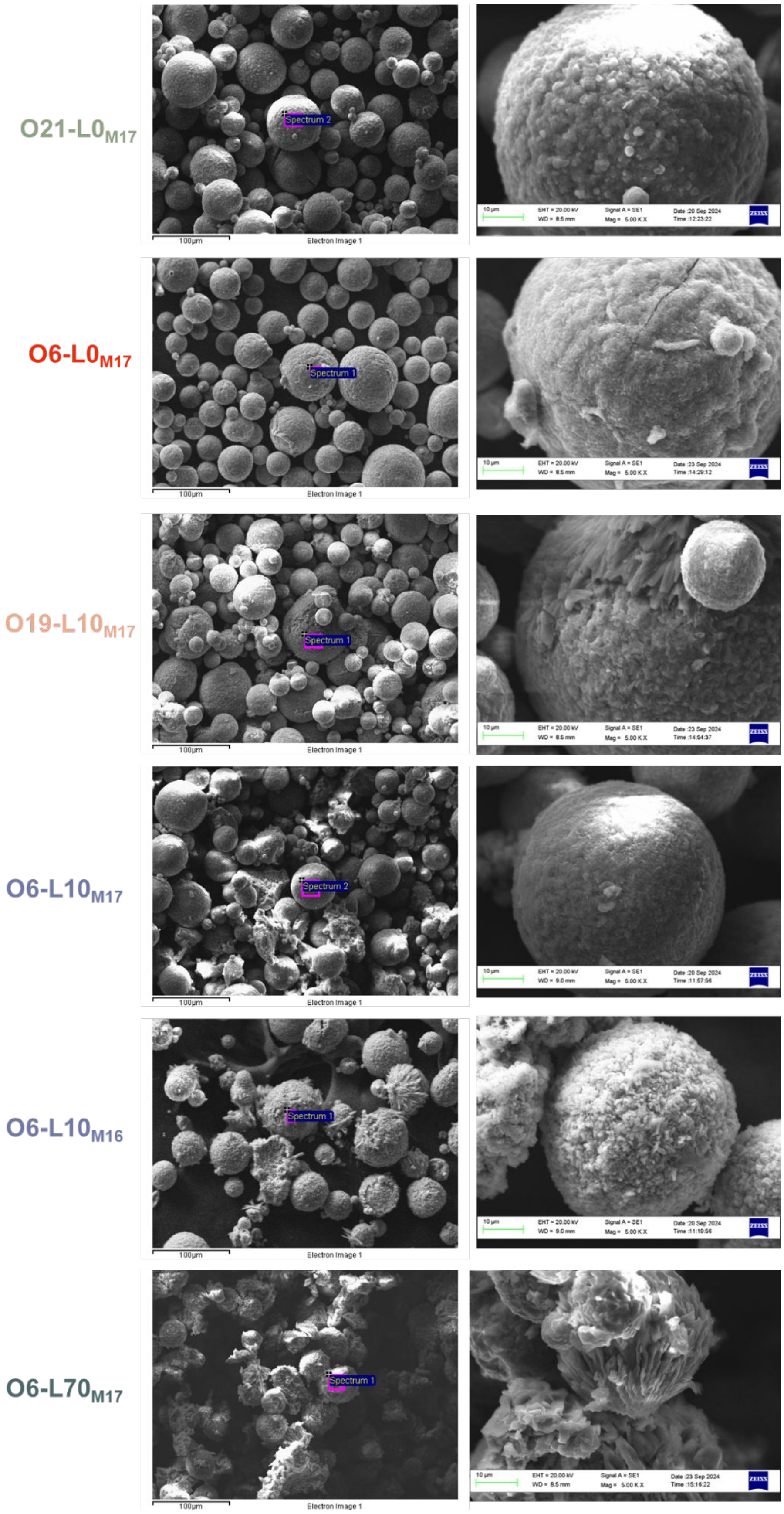

Figure S13. SEM characterization of samples O6-L0, O19-L10, O6-L10, O6-L70, O21-L0 and sample O6-L10$_{M16}$.

Table S3. Average P/V atomic ratio of samples O6-L0, O19-L10, O6-L10, O6-L70, O21-L0, and sample O6-L10$_{M16}$ obtained with EDX multipoint measurements. [a] P/V ratio of VHP precursors evaluated by XRF.

Catalyst	precursors[a] P/V atomic ratio	Calcined samples P/V atomic ratio
**O21-L0$_{M17}$**	1.15	1.19
**O6-L0$_{M17}$**	1.15	1.15
**O19-L10$_{M17}$**	1.15	1.21
**O6-L10$_{M17}$**	1.15	1.18
**O6-L10$_{M16}$**	1.20	1.31
**O6-L70$_{M17}$**	1.15	1.20

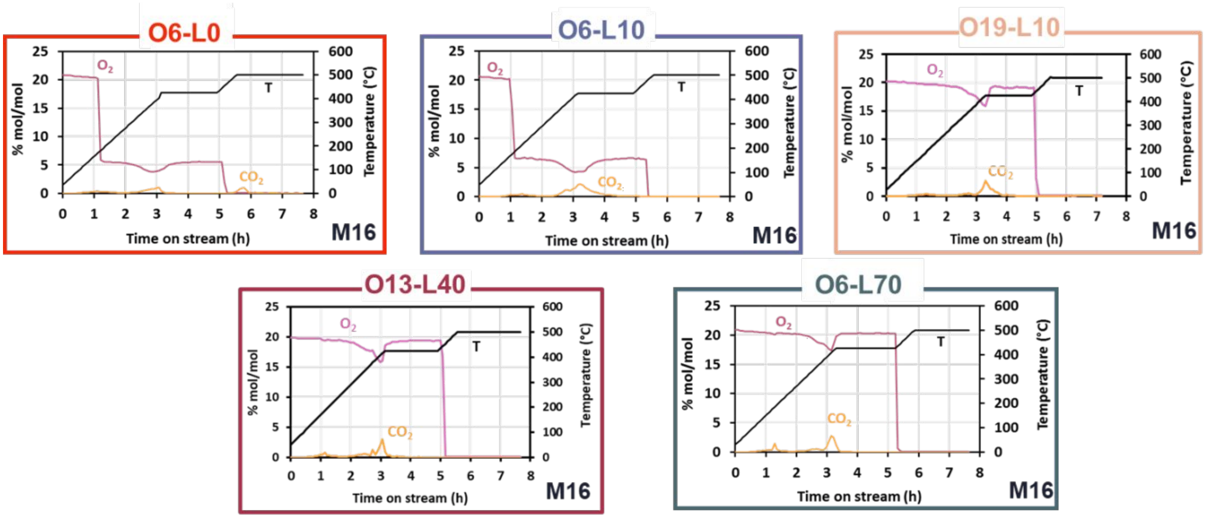

Figure S14. CO_2 evolution (orange) and oxygen consumption (yellow) during calcination of samples O19-L10$_{M16}$ (light orange- N_2:O_2:H_2O 71:19:10), O13-L40$_{M16}$ (purple- N_2:O_2:H_2O 47:13:40), O6-L0$_{M16}$ (red- N_2:O_2:H_2O 94:6:0), O6-L10$_{M16}$ (violet- N_2:O_2:H_2O 84:6:10), O6-L70$_{M16}$ (light blue- N_2:O_2:H_2O 24:6:70).

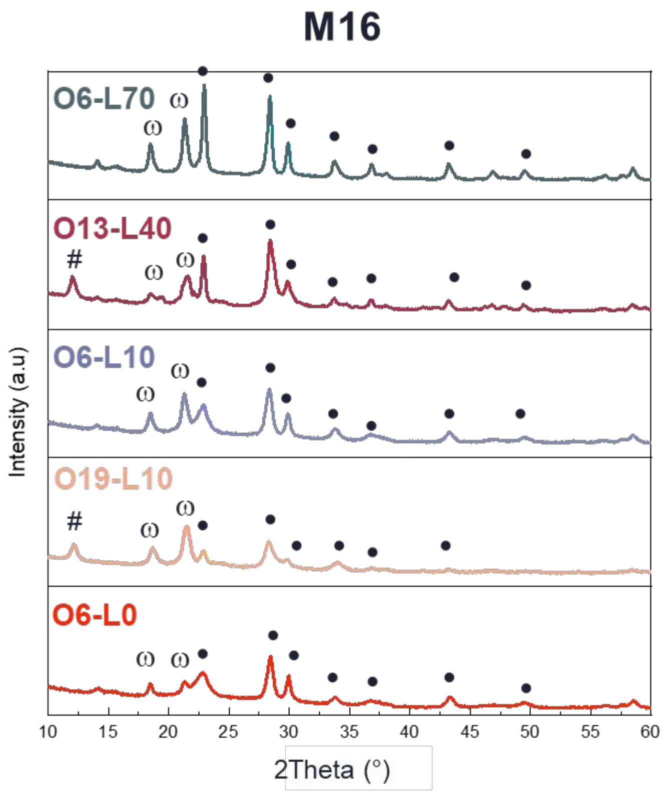

Figure S15. XRD pattern of samples O19-L10$_{M16}$ (light orange- N_2:O_2:H_2O 71:19:10), O13-L40$_{M16}$ (purple- N_2:O_2:H_2O 47:13:40), O6-L0$_{M16}$ (red- N_2:O_2:H_2O 94:6:0), O6-L10$_{M16}$ (violet- N_2:O_2:H_2O 84:6:10), O6-L70$_{M16}$ (light blue- N_2:O_2:H_2O 24:6:70) after calcination. Symbols: ω= ω-$VOPO_4$; ● = VPP.

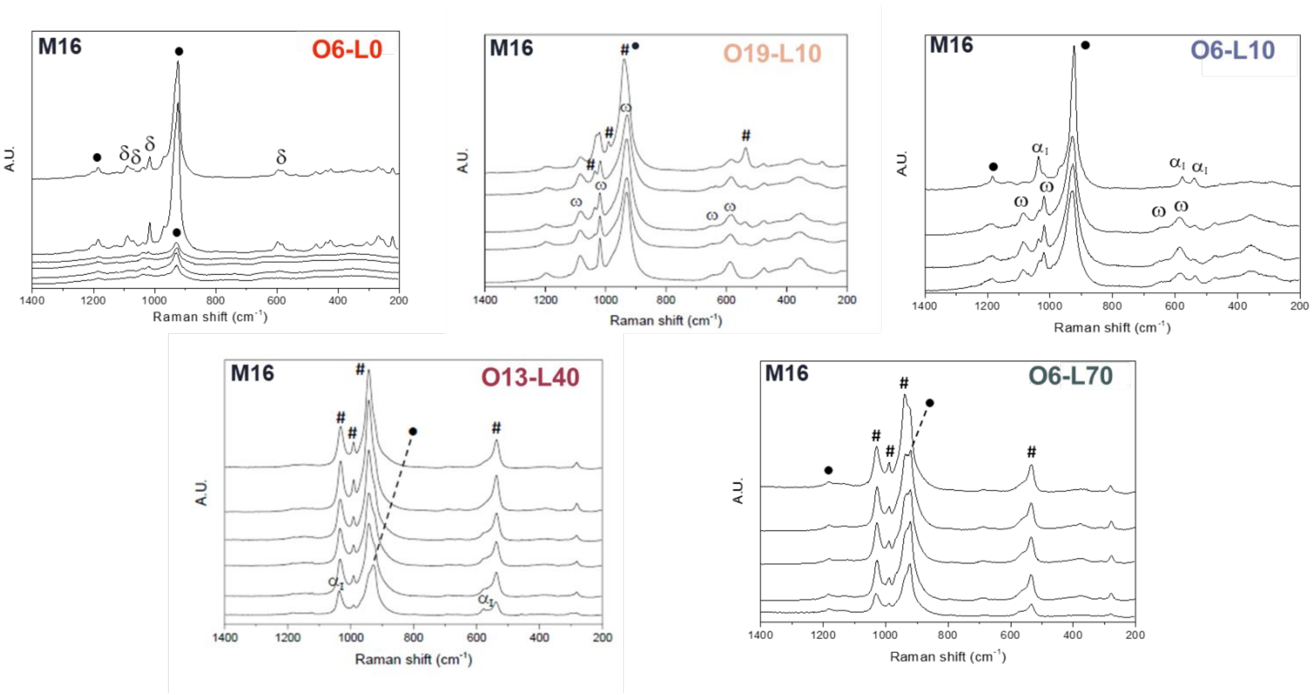

Figure S16. Raman spectra of samples O19-L10$_{M16}$ (light orange- N_2:O_2:H_2O 71:19:10), O13-L40$_{M16}$ (purple- N_2:O_2:H_2O 47:13:40), O6-L0$_{M16}$ (red- N_2:O_2:H_2O 94:6:0), O6-L10$_{M16}$ (violet- N_2:O_2:H_2O 84:6:10), O6-L70$_{M16}$ (light blue- N_2:O_2:H_2O 24:6:70) after calcination. Symbols: ω= ω-$VOPO_4$; δ= δ-$VOPO_4$ ~~α_1~~ α_I = ~~α_1~~ α_I-$VOPO_4$, ● = VPP, # =

VOPO$_4$•2H$_2$O. For each sample, various spectra were collected from different surface spots of possible interest (different shapes, colors, etc.).

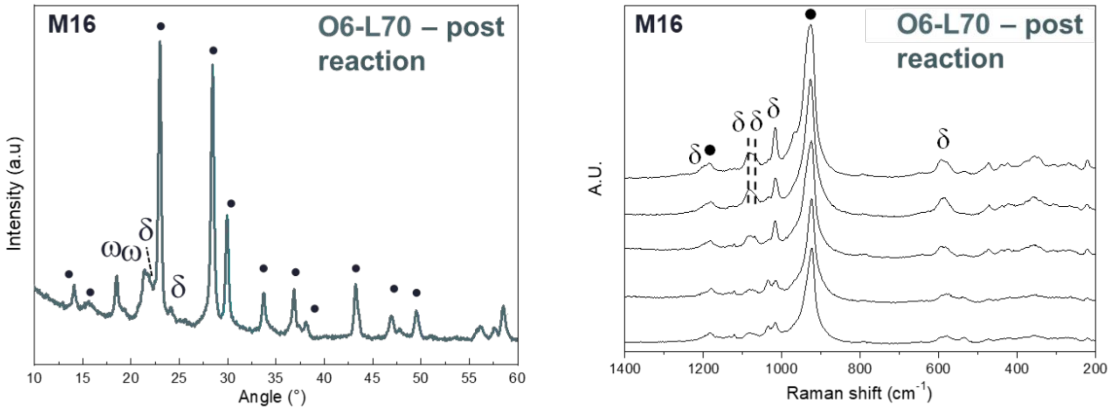

Figure S17. XRD analysis (left) and Raman spectra (right) of sample O6-L70$_{M16}$ after reaction. Symbols: ω= ω-VOPO$_4$; δ= δ-VOPO$_4$, ●= VPP. Various Raman spectra were collected from different surface spots of possible interest (different shapes, colors, etc.).

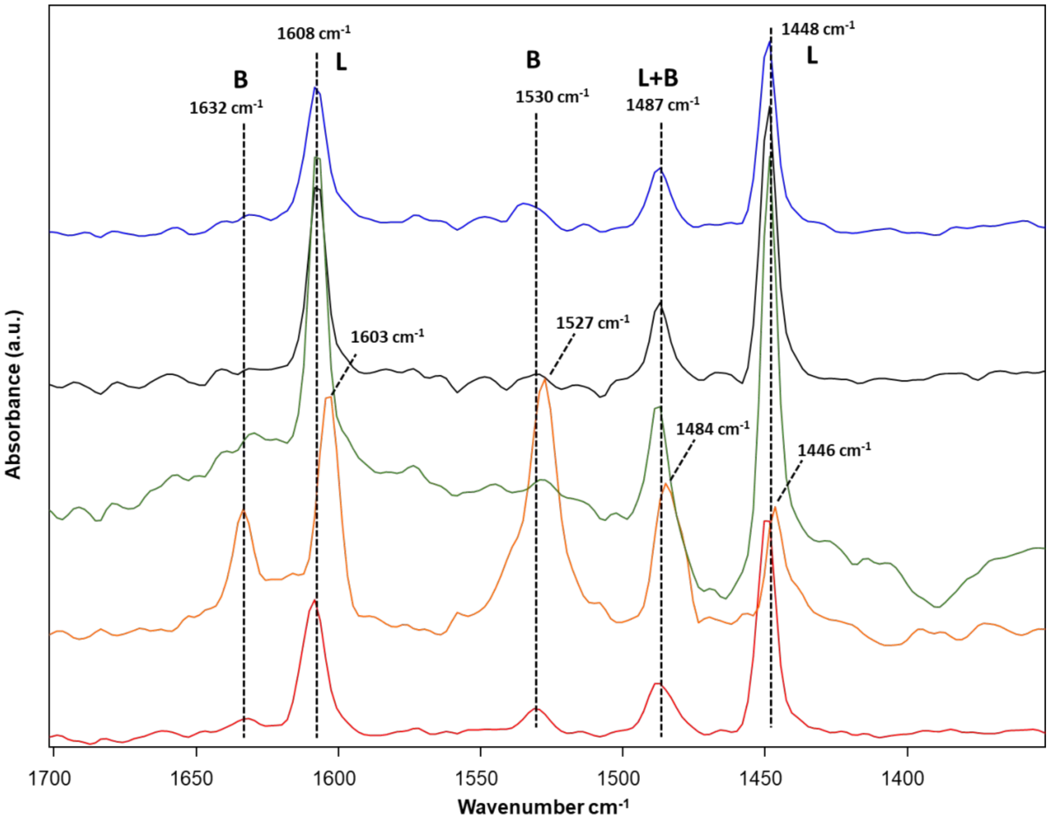

Figure S18. DRIFT pyridine spectra of calcined samples. From the top to the bottom: O19-L10$_{M17}$ (blue), O6-L0$_{M17}$ (black), O6-L10$_{M17}$ (green), O6-L70$_{M17}$ (orange), O6-L10$_{M16}$ (red).

Table S4. Comparison of the catalytic performance of O6-L10$_{M16}$ O19-L10$_{M17}$ with those reported in the literature.

Ref.	%butane (v/v)	GHSV (h^{-1})	T (°C)	Conv.	Sel.	Yield
**O6-L10$_{M16}$**	1.7	2700	400	51%	73%	37%
**O6-L10$_{M17}$**	1.7	2700	400	41%	72%	30%
[1]	1.4	1200	400-420	65-89%	71-84%	54-64%
[2]	1.7	5000	380	72%	42%	30%
[3]	1.7	2000	400	50%	61%	31%
[4]	1.7	2000	400	87%	64%	56%
[5]	1.4	2000	400	88%	64%	56%
[6]	1.5	2000	400	92%	61%	56%
[7]	1.3	2000	420	94%	66%	62%

(1) Zhang, Y.; Zhang, R.; Dong, J.; Wu, Y.; Luo, C.; Zhang, H.; Zhu, S.; Liu, R. The Critical Role of Steam during Activation Process on the Catalytic Performance of VPO for n-Butane Selective Oxidation to Maleic Anhydride. *J Catal* **2022**, *416*, 157–169. https://doi.org/10.1016/j.jcat.2022.11.004.

(2) Nguyen Dinh, M. T.; Nguyen, T. L.; Phan, M. D.; Nguyen Dinh, L.; Truong, Q. D.; Bordes-Richard, E. Control of the Crystal Morphology of VOHPO4·0.5H2O Precursors Prepared via Light Alcohols-Assisted Solvothermal Synthesis and Influence on the Selective Oxidation of n-Butane. *J Catal* **2019**, *377*, 638–651. https://doi.org/10.1016/j.jcat.2019.08.004.

(3) Weng, W.; Al Otaibi, R.; Alhumaimess, M.; Conte, M.; Bartley, J. K.; Dummer, N. F.; Hutchings, G. J.; Kiely, C. J. Controlling Vanadium Phosphate Catalyst Precursor Morphology by Adding Alkane Solvents in the Reduction Step of $VOPO_4·2H_2O$ to $VOHPO_4·0.5H_2O$. *J Mater Chem* **2011**, *21* (40), 16136–16146. https://doi.org/10.1039/c1jm12456k.

(4) Xu, J.; Li, N.; Li, X.; Ji, R.; Yan, X.; Huang, R.; Li, C. Role of Regulating Synthetic Solvents in Enhancing the Catalytic Performance of VPO Catalysts for N-Butane Oxidation to Maleic Anhydride. *Chemical Engineering Journal* **2024**, *496*. https://doi.org/10.1016/j.cej.2024.153635.

(5) Zhang, X.; Wang, H.; Gou, L.; Li, L.; Duan, A.; Cao, Z. Reaction Behaviors and Crystal Transformation of Industrial Vanadium-Phosphorus-Oxygen (VPO) Catalysts for n-Butane Oxidation. *ACS Omega* **2021**, *6* (36), 23558–23563. https://doi.org/10.1021/acsomega.1c03652.

(6) He, B.; Li, Z.; Zhang, H.; Dai, F.; Li, K.; Liu, R.; Zhang, S. Synthesis of Vanadium Phosphorus Oxide Catalysts Assisted by Deep-Eutectic Solvents for n-Butane Selective Oxidation. *Ind Eng Chem Res* **2019**, *58* (8), 2857–2867. https://doi.org/10.1021/acs.iecr.8b06010.

(7) Zhang, T.; Zhang, R.; Zhang, Y.; Xie, Z.; Li, Y.; Wu, H.; Dai, F.; Liu, R. Phosphoric Acid: A Key Role in Control of Structure and Properties of Vanadium Phosphorus Oxide Catalysts During Synthesis. *ChemistrySelect* **2021**, *6* (4), 513–521. https://doi.org/10.1002/slct.202003236.
